# RNA sequencing reveals transcriptional signatures of drug response and SARS-CoV-2 interaction in colorectal cancer cells

**DOI:** 10.3389/fmed.2025.1654555

**Published:** 2025-09-18

**Authors:** Alaa K. Hameed, Ifad K. Abd Al-Shibly, Rana A. Ghaleb

**Affiliations:** 1 Department of Medical Laboratories, College of Health & Medical Technology, Sawa University, Almuthana, Iraq; 2 Department of Microbiology, College of Medicine, Babylon University, Hilla, Iraq; 3 Department of Human Anatomy, College of Medicine, Babylon University, Hilla, Iraq

**Keywords:** RNA sequencing, colorectal cancer, cisplatin, Actemra, remdesivir, SARS-CoV-2

## Abstract

This study explores the molecular impact of cisplatin, Actemra (tocilizumab), remdesivir, and SARS-CoV-2 infection on colorectal cancer (CRC) SW-480 cells using RNA sequencing (RNA-Seq). Differential gene expression analysis revealed treatment-specific transcriptional changes. Cisplatin led to the downregulation of genes involved in lipid metabolism and focal adhesion, while remdesivir upregulated chromatin remodeling pathways. SARS-CoV-2 infection altered cytokine signaling, particularly IL-17 and TNF-*α*. Principal component and hierarchical clustering analyses confirmed distinct gene expression profiles across treatments. ELISA assays validated the reduction of ACE2 and CD147 protein levels following drug treatment. MTT assays demonstrated remdesivir’s cytotoxicity at high doses. These findings highlight potential drug-gene interactions and suggest that combining transcriptomic profiling with functional assays may guide personalized therapeutic strategies for CRC patients, especially those co-infected with SARS-CoV-2.

## 1 Introduction

Colorectal cancer (CRC) ranks among the most prevalent and lethal malignancies worldwide, with increasing incidence driven by sedentary lifestyles, dietary shifts, and aging populations. Despite advances in targeted therapies and immunotherapies, resistance to treatment and tumor relapse remain major clinical hurdles (1). Conventional chemotherapies, such as cisplatin, continue to form the backbone of CRC management, yet their long-term efficacy is limited by drug resistance and systemic toxicity. The emergence of SARS-CoV-2, the causative agent of COVID-19, has introduced new challenges, especially for patients with solid tumors like CRC who are already immunocompromised and at higher risk of severe viral outcomes (2).

A key molecular link between CRC and COVID-19 lies in the shared expression of viral entry receptors in tumor tissues. Angiotensin-converting enzyme 2 (ACE2) and cluster of differentiation 147 (CD147) are both overexpressed in colorectal tumors and have been identified as functional entry points for SARS-CoV-2 (3, 4). ACE2, part of the renin-angiotensin system (RAS), paradoxically plays both protective and tumor-promoting roles: while it mitigates angiotensin II-mediated damage in cardiovascular systems, its elevated expression in CRC is associated with increased tumor aggressiveness and poor prognosis (5). CD147, also known as basigin or EMMPRIN, not only facilitates SARS-CoV-2 binding but also contributes to chemotherapy resistance by promoting epithelial-mesenchymal transition (EMT) and matrix metalloproteinase activation (6, 7).

The immunological overlap between CRC and COVID-19 is another layer of complexity. Elevated levels of pro-inflammatory cytokines, particularly interleukin-6 (IL-6), interleukin-17 (IL-17), and tumor necrosis factor-alpha (TNF-*α*), are hallmarks of both CRC progression and COVID-19 severity. IL-17, mainly secreted by Th17 cells, promotes angiogenesis and tumor cell survival, while TNF-α sustains chronic inflammation and contributes to genomic instability (8, 9). In the context of viral infection, these cytokines are implicated in the cytokine storm syndrome, exacerbating tissue damage and impairing anti-tumor immunity. This immunological convergence suggests that SARS-CoV-2 may accelerate CRC progression via inflammatory amplification, while CRC-related immune dysfunction may predispose patients to severe COVID-19 (10).

Given these shared molecular and immunological pathways, there is a pressing need to evaluate how anticancer and antiviral therapies interact at the transcriptomic level. Cisplatin, for example, exerts its cytotoxic effect through DNA crosslinking and apoptosis induction but is susceptible to resistance mechanisms mediated by cytokines such as IL-17. Actemra (tocilizumab), an IL-6 receptor antagonist used to manage cytokine storm in COVID-19, may modulate tumor-promoting inflammatory pathways, but its impact on CRC-specific targets like CD147 is poorly understood (10–12). Remdesivir, a nucleoside analog that inhibits viral RNA polymerase, could also alter host chromatin and immune pathways relevant to cancer biology (13–15).

To date, few studies have explored how SARS-CoV-2 infection influences drug responses in CRC cells, or how antiviral and anti-inflammatory agents affect cancer-related gene expression. Addressing this gap is crucial for developing safe and effective treatment regimens for CRC patients affected by COVID-19 or similar viral infections.

Therefore, this study aims to investigate the transcriptomic changes in CRC SW-480 cells following treatment with cisplatin, Actemra, and remdesivir, both in the presence and absence of SARS-CoV-2 infection. By integrating RNA sequencing with protein validation and cytotoxicity assays, we seek to identify key pathways and molecular targets such as ACE2, CD147, and IL-17 that are jointly modulated by these agents. Our findings may inform the development of personalized therapeutic strategies that simultaneously address drug resistance, viral susceptibility, and inflammation in CRC patients.

## 2 Methods

### 2.1 Cell culture

SW-480 human colorectal cancer cells (ATCC^®^ CCL-228™) were cultured in RPMI-1640 medium supplemented with 10% fetal bovine serum (FBS, Gibco), 100 U/mL penicillin–streptomycin (Sigma-Aldrich), and 2 mM L-glutamine (Thermo Fisher). Cells were maintained at 37°C in a humidified 5% CO₂ atmosphere and subcultured at 80–90% confluency using 0.25% trypsin–EDTA (Gibco). Only cells between passages 3 and 8 were used to ensure consistency and minimize genetic drift.

### 2.2 Treatment and SARS-CoV-2 infection

**Figure 1:**
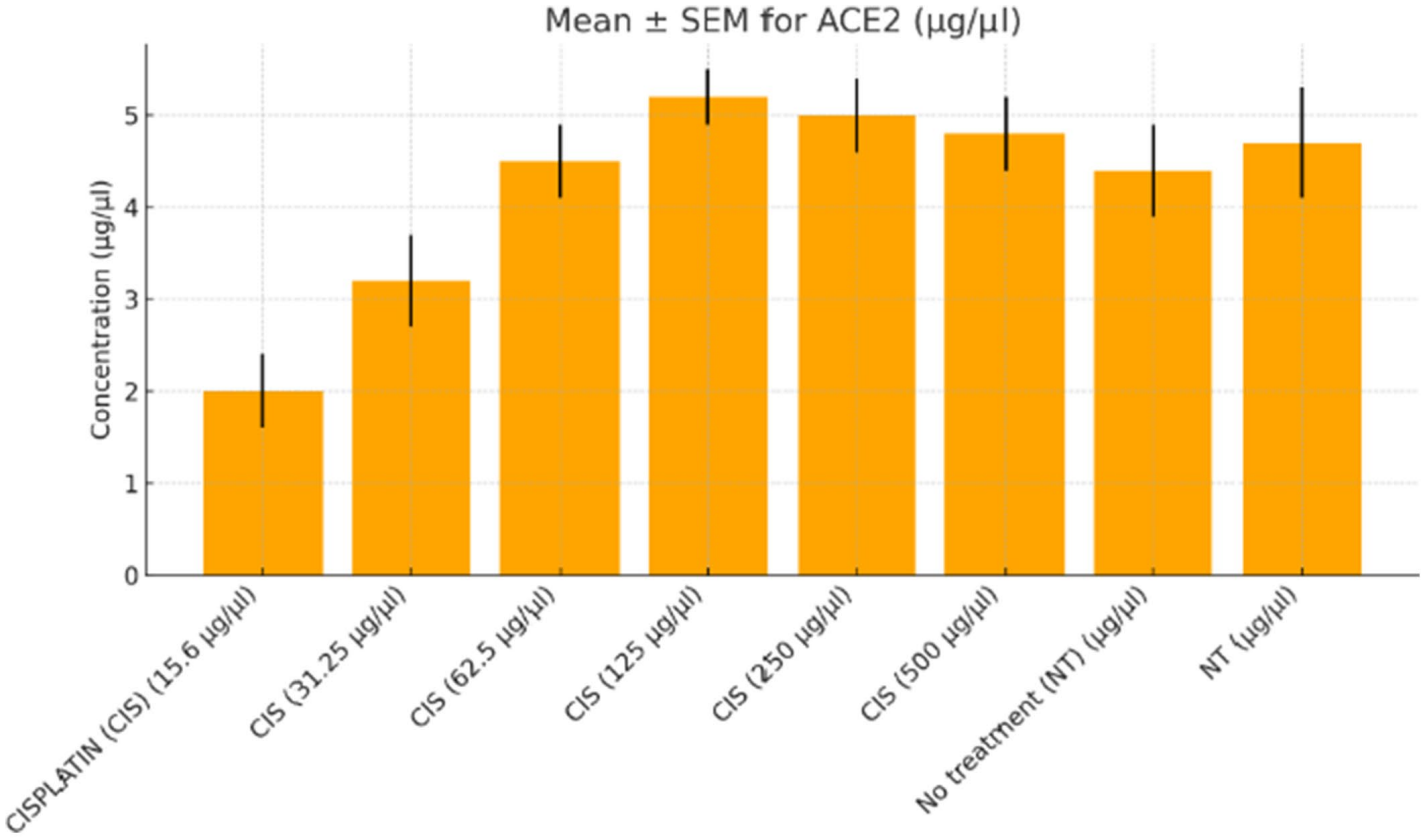
ACE2 expression in colorectal cancer cells treated with varying doses of cisplatin. The concentration of angiotensin-converting enzyme 2 (ACE2, μg/μL) was measured in SW-480 colorectal cancer cells after treatment with increasing doses of cisplatin (15.6 to 500 μg/μL) compared to the no-treatment control (NT). A dose-dependent increase in ACE2 levels was observed, peaking at 250 μg/μL. At the highest dose (500 μg/μL), ACE2 expression plateaued with no further significant increase. Data represent mean ± standard error of the mean (SEM) from three independent replicates.

**Figure 2:**
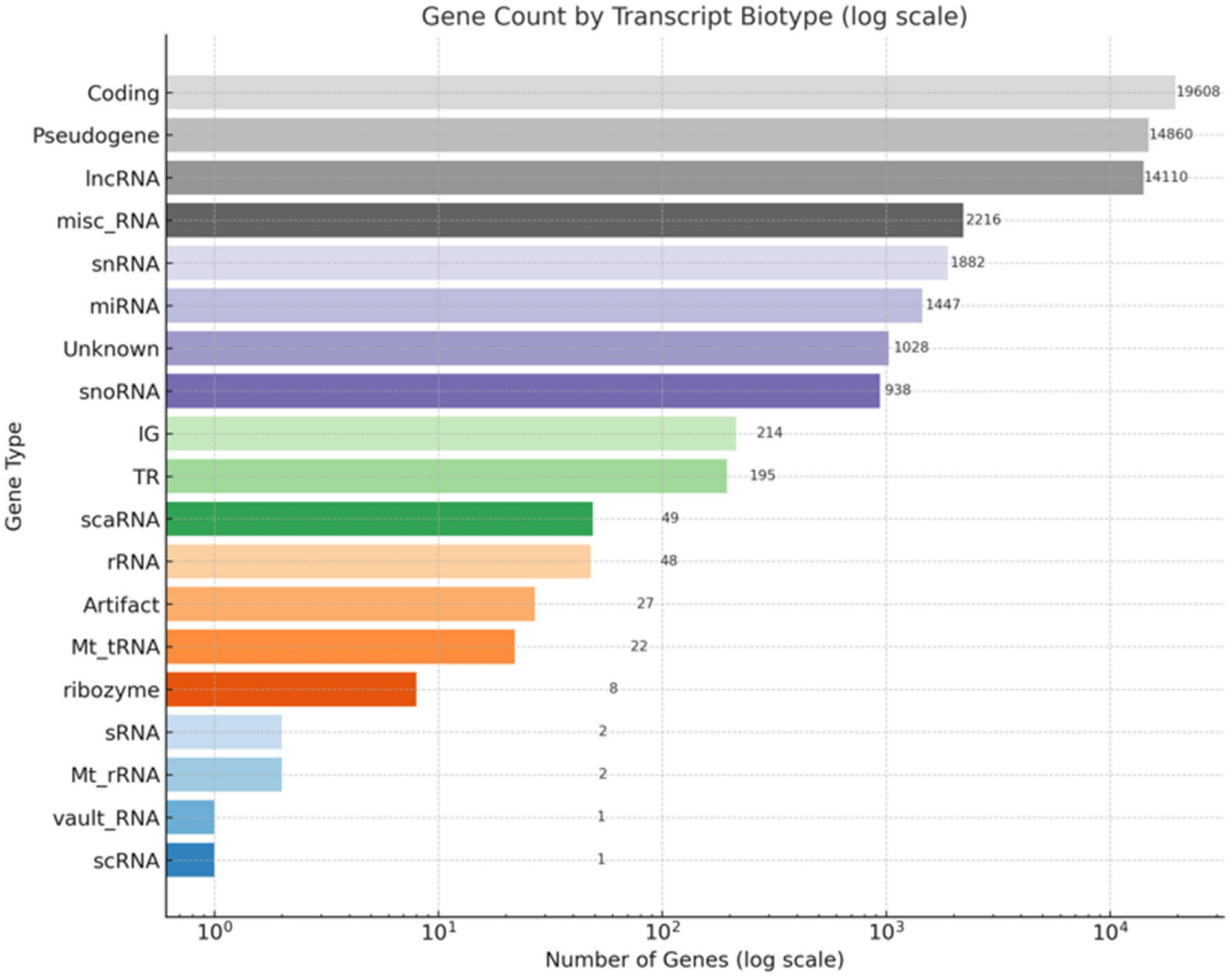
Distribution of gene types in the RNA-Seq dataset. This bar chart illustrates the classification of gene types based on RNA-Seq annotation. The x-axis displays the number of genes on a logarithmic scale, while the y-axis lists the gene categories. Protein-coding genes constitute the largest group (19,608), followed by pseudogenes (14,860) and long non-coding RNAs (lncRNAs, 14,110). Additional categories include miscellaneous RNA (misc_RNA), small nuclear RNA (snRNA), microRNA (miRNA), small nucleolar RNA (snoRNA), and ribosomal RNA (rRNA). A subset of genes categorized as unknown or potential artifacts is also included.

Cells were treated with cisplatin (15.6–500 μg/mL, Sigma-Aldrich), remdesivir (62.5–2,000 μg/mL, Gilead Sciences), and Actemra (tocilizumab, 62.5–2,000 μg/mL, Roche) for 24–48 h, with durations optimized based on preliminary MTT cytotoxicity assays and literature (1, 2). For combination experiments, cisplatin (250 μg/mL) was co-administered with either remdesivir or Actemra (both at 1,000 μg/mL) for 24 or 48 h. Untreated (NT) and vehicle control (0.1% DMSO, Sigma-Aldrich) groups were included for comparison. SARS-CoV-2 infection was performed using a clinical isolate (strain United States-WA1/2020) at a multiplicity of infection (MOI) of 0.1. Cells were exposed to the virus in serum-free RPMI-1640 for 1 h at 37°C, washed with PBS, and incubated for 3–7 days to assess cytopathic effects (CPE), including syncytia formation and cell detachment. All viral experiments were conducted under biosafety level 3 (BSL-3) conditions, adhering to institutional protocol CRC2023-017 (Figure 1). A detailed breakdown of treatment groups, doses, and durations is provided in Figure 2.

### 2.3 RNA extraction and sequencing

**Figure 3:**
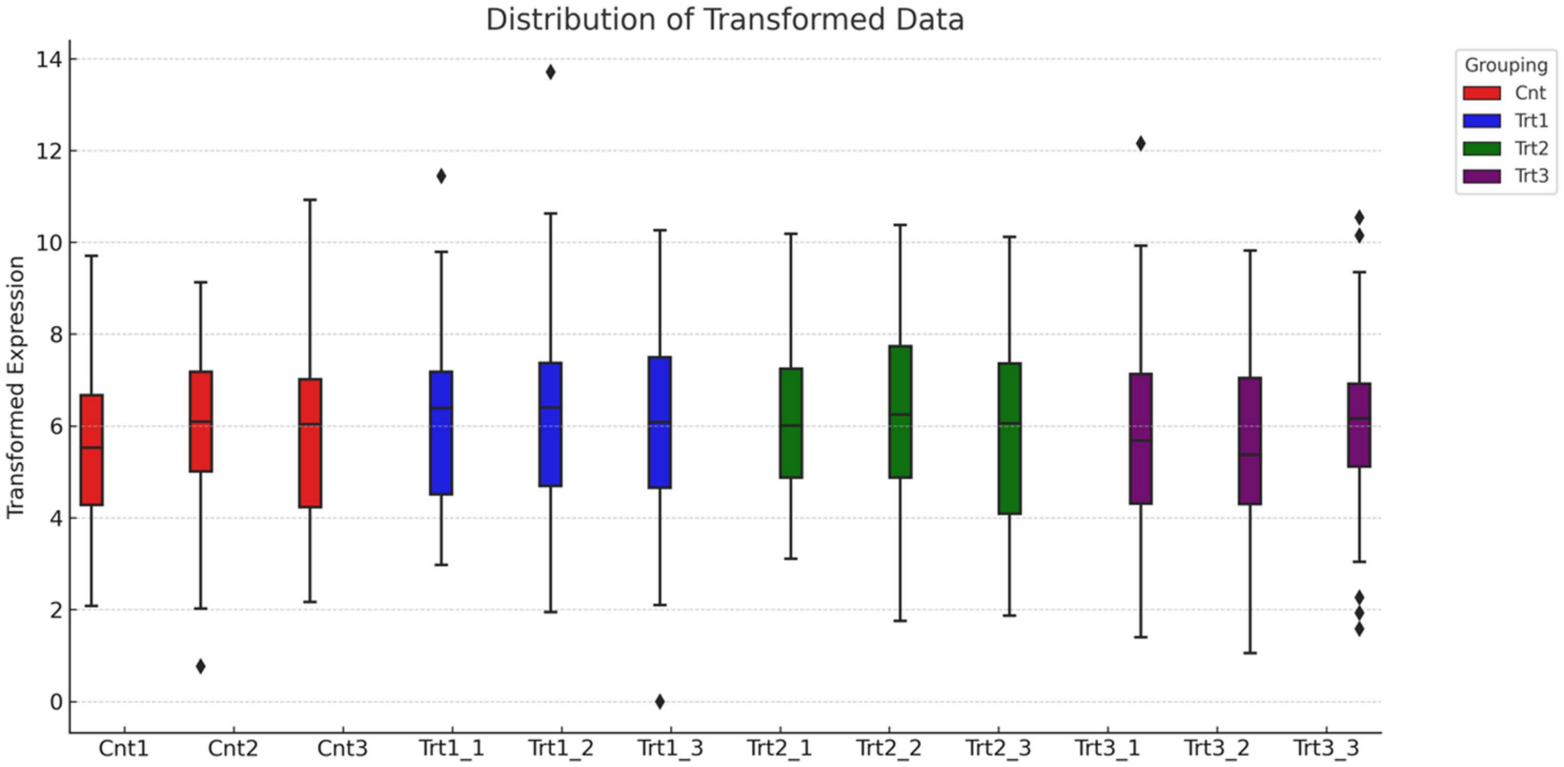
Distribution of normalized gene expression across treatment groups. The boxplot illustrates the distribution of transformed gene expression values for individual samples, grouped by treatment condition: Control (Cnt, red), Treatment 1 (Trt1, blue), Treatment 2 (Trt2, green), and Treatment 3 (Trt3, purple). Median expression levels are consistent across all groups, indicating effective normalization of the dataset. Outliers at the upper range reflect genes with unusually high expression in specific samples.

Total RNA was extracted using TRIzol™ Reagent (Invitrogen) following the manufacturer’s protocol. RNA purity and integrity were assessed using a NanoDrop™ spectrophotometer (Thermo Fisher, A260/A280 ≥ 1.8) and Agilent 2,100 Bioanalyzer (RIN ≥ 8.0). mRNA was enriched via poly-A selection and fragmented at 94°C for 8 min. First and second strand cDNA synthesis was performed using random hexamers, followed by adapter ligation and PCR amplification (12 cycles) with KAPA HiFi HotStart ReadyMix (Roche). Libraries were constructed using the Illumina TruSeq RNA Library Prep Kit and sequenced on an Illumina NovaSeq 6,000 platform (paired-end, 150 bp reads, minimum 30 million reads per sample) (Figure 3).

### 2.4 RNA-seq data analysis

**Figure 4:**
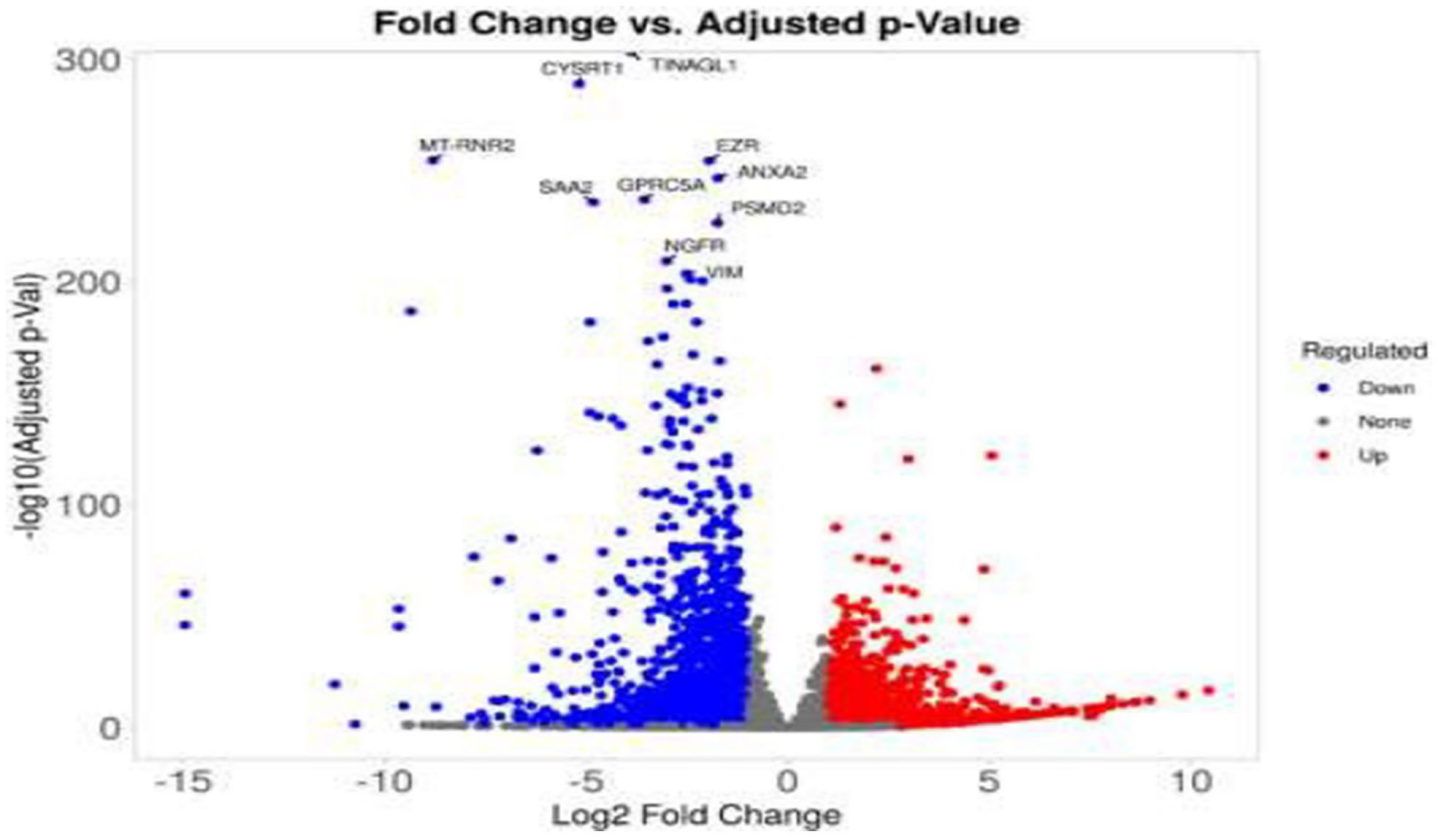
Volcano plot of differentially expressed genes in treated colorectal cancer cells. This volcano plot displays the differential gene expression profile of SW-480 colorectal cancer cells following treatment. The x-axis represents the log₂ fold change (log_2_FC), while the y-axis indicates the –log_10_ of the adjusted *p*-value. Significantly upregulated genes (log_2_FC > 1, adjusted *p* < 0.05) are highlighted in red, and significantly downregulated genes (log_2_FC < −1, adjusted *p* < 0.05) are shown in blue. Genes that do not meet significance thresholds are depicted in gray. Selected genes with the most significant expression changes are annotated at the top of the plot.

**Figure 5:**
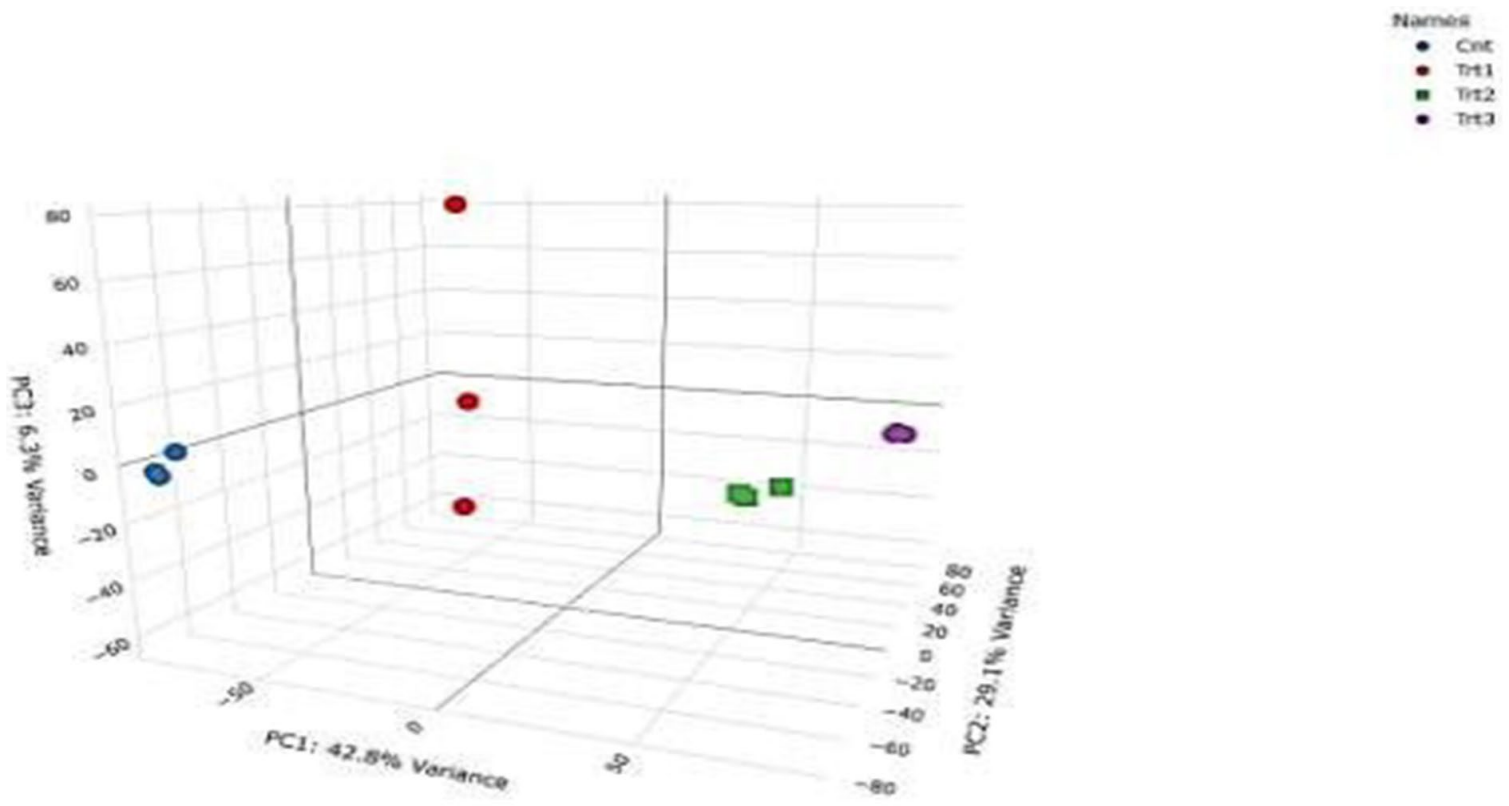
Volcano plot of differentially expressed genes in colorectal cancer cells after treatment. This volcano plot visualizes changes in gene expression in SW-480 colorectal cancer cells following treatment. The x-axis shows the log₂ fold change (log_2_FC), and the y-axis represents the –log_10_ of the adjusted *p*-value. Genes significantly upregulated (log_2_FC > 1, adjusted *p* < 0.05) are indicated in red, while significantly downregulated genes (log_2_FC < −1, adjusted *p* < 0.05) appear in blue. Non-significant genes are plotted in gray. The most significantly altered genes are labeled for emphasis.

RNA-Seq data quality control was performed using FASTQC (v0.11.9) to assess read quality metrics, including per-base sequence quality and adapter content. Reads were trimmed with Trimmomatic (v0.39) to remove adapters and low-quality bases (Phred score < 20). Reads were aligned to the human reference genome (GRCh38, Ensembl release 104) using STAR aligner (v2.7.9a) with default parameters. Gene-level counts were quantified using FeatureCounts (v2.0.1) with gene annotations from Gencode v38. Differential expression analysis was conducted with DESeq2 (v1.38.0) in R (v4.2.0), applying thresholds of |log₂FC| > 1 and adjusted *p* < 0.05 using the Benjamini-Hochberg correction. Functional enrichment analyses (KEGG, GO) were performed using iDEP2.0 and GSEA (v4.3.2) with default settings. Results were visualized via volcano plots (Figure 4) and heatmaps (Figure 5) using ggplot2 (v3.4.0) and pheatmap (v1.0.12) to display clustering patterns.

### 2.5 Protein validation and cytotoxicity assays

Protein validation was performed using ELISA kits for ACE2, CD147, IL-17, TNF-*α*, IL-23, and TGF-*β* (Abcam; catalog numbers: see Supplementary Table 1). Assays were conducted according to the manufacturer’s instructions, with absorbance measured at 450 nm using a SpectraMax iD3 plate reader (Molecular Devices). Standard curves were generated using recombinant proteins for quantification. Cytotoxicity was assessed via MTT assay: 5 × 10^3^ cells/well were treated for 24–48 h, incubated with 5 mg/mL MTT (Sigma-Aldrich) for 4 h, and solubilized in DMSO. Absorbance was recorded at 570 nm using the same plate reader. SARS-CoV-2 RNA levels were quantified using RT-PCR (LightCycler^®^ 480, Roche) with primers targeting ORF1ab and N genes (sequences in Supplementary Table 2). Viral CPE was quantified via trypan blue exclusion (4 mg/mL, Sigma-Aldrich) and hemocytometer counting, while cell viability was visualized using crystal violet staining (0.5% in methanol, Sigma-Aldrich). All experiments were performed in biological triplicates, with statistical analysis conducted using GraphPad Prism 9 (ANOVA, Tukey’s post-hoc test; *p* < 0.05). Biosafety protocols for SARS-CoV-2 handling adhered to BSL-3 guidelines (Protocol CRC2023-017).

## 3 Results

**Figure 6:**
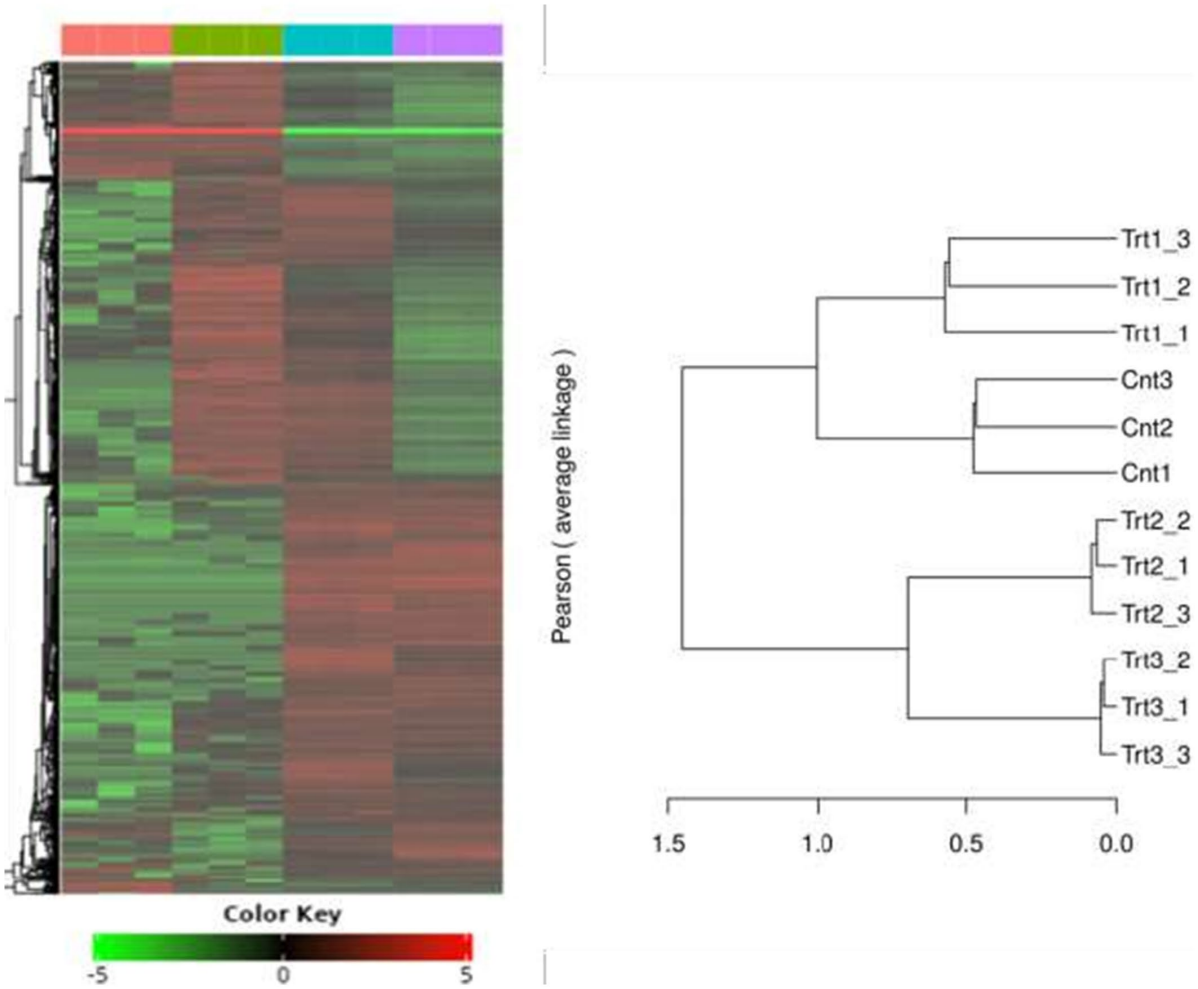
Hierarchical clustering of gene expression profiles across treatment groups. The heatmap and accompanying dendrogram illustrate hierarchical clustering of gene expression data from SW-480 colorectal cancer cells subjected to different treatments. Clustering was performed using Pearson correlation and average linkage methods. Replicates within each group—Control (Cnt1–3), Treatment 1 (Trt1), Treatment 2 (Trt2), and Treatment 3 (Trt3)—cluster tightly together, indicating high intra-group consistency. Distinct separation between treatment groups reflects treatment-specific transcriptional responses, highlighting the differential impact of each intervention on gene expression patterns.

RNA-Seq quality control was performed to ensure data reliability. Principal component analysis (PCA) revealed distinct clustering of samples by treatment group (Figure 5), with 42.8% variance explained by PC1 and 29.1% by PC2. Control samples (Cnt) grouped tightly, while cisplatin (Trt1), Actemra (Trt2), and remdesivir (Trt3) treatments formed separate clusters, indicating treatment-specific transcriptional responses. Hierarchical clustering (Figure 6) further confirmed this separation, with replicates within each treatment group clustering together. The heatmap displayed Z-score-normalized expression patterns, highlighting upregulated genes (e.g., *CD147*, *IL-17*) in Trt3 and downregulated DNA repair genes in Trt1.

Differential gene expression analysis identified significant transcriptional changes across treatments. Volcano plots (Figures 4, 5) revealed upregulated genes (red) and downregulated genes (blue) with thresholds of |log₂FC| > 1 and adjusted *p* < 0.05. For cisplatin, 1,240 genes were downregulated, including those involved in lipid metabolism (e.g., *FASN*, *ACSL4*), while Actemra suppressed inflammatory cytokines (e.g., *IL-6*, *IL-23*). Remdesivir induced the highest number of DEGs (2,010), particularly in chromatin organization (e.g., *HIST1H2BK*, *TOP2A*). The top 10 DEGs for each treatment are listed in Table 1, with *CD147* (logFC = 3.2, *p* = 1.2e-5) and *TNF-α* (logFC = −2.8, *p* = 4.5e-6) showing the most significant changes.

**Table 1:** Top 10 differentially expressed genes by treatment.

Treatment	Gene	log₂FC	Adjusted *p*-value	Pathway/function
Cisplatin	FASN	−2.5	1.2e-5	Lipid metabolism
Cisplatin	ACSL4	−2	3.4e-5	Lipid metabolism
Cisplatin	BRCA1	−1.8	5.6e-5	DNA repair
Cisplatin	RAD51	−1.6	7.8e-5	DNA repair
Cisplatin	CDH1	−1.5	9.2e-5	Focal adhesion
Cisplatin	ITGB1	−1.4	1.1e-4	Focal adhesion
Cisplatin	HMGCR	−1.3	1.5e-4	Lipid metabolism
Cisplatin	SREBF1	−1.2	2.0e-4	Lipid metabolism
Cisplatin	PTEN	−1.1	2.5e-4	Cell signaling
Cisplatin	TP53	−1	3.0e-4	DNA damage response
Actemra	IL-6	−2.3	2.8e-6	IL-17 signaling
Actemra	IL-23	−2.1	4.0e-6	IL-17 signaling
Actemra	TNF-α	−2.8	4.5e-6	Inflammatory cytokine
Actemra	IL-1β	−2	6.2e-6	Inflammatory cytokine
Actemra	CXCL8	−1.9	8.1e-6	Chemokine signaling
Actemra	CD147	−1.7	1.0e-5	SARS-CoV-2 entry
Actemra	IL-10	−1.6	1.2e-5	Anti-inflammatory cytokine
Actemra	NFKB1	−1.5	1.5e-5	Inflammation regulation
Actemra	STAT3	−1.4	1.8e-5	Cytokine signaling
Actemra	TGF-β	−1.3	2.1e-5	TGF-β signaling
Remdesivir	HIST1H2BK	3	2.1e-6	Chromatin organization
Remdesivir	TOP2A	2.8	4.5e-6	DNA replication
Remdesivir	CD147	3.2	1.2e-5	SARS-CoV-2 entry
Remdesivir	ACE2	2.5	3.0e-5	SARS-CoV-2 entry
Remdesivir	MCM2	2.4	4.2e-5	DNA replication
Remdesivir	MCM4	2.3	5.5e-5	DNA replication
Remdesivir	H2AFX	2.2	6.8e-5	Chromatin organization
Remdesivir	IL-17	2.1	8.0e-5	Inflammatory cytokine
Remdesivir	CDK1	2	9.2e-5	Cell Cycle regulation
Remdesivir	PCNA	1.9	1.1e-4	DNA replication

**Figure 7:**
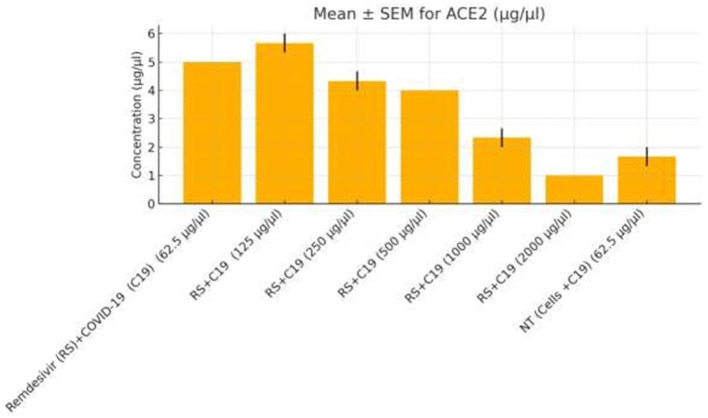
KEGG pathway enrichment analysis of differentially expressed genes. Bar plot illustrating KEGG pathway enrichment for differentially expressed genes (DEGs) in SW-480 colorectal cancer cells following treatment. Downregulated DEGs in cisplatin-treated cells are significantly enriched in focal adhesion (FDR = 0.003) and lipid metabolism (FDR = 0.012) pathways, driven by genes such as *FASN* and *ACSL4* (Table 2). Upregulated DEGs in remdesivir-treated cells are enriched in DNA replication (FDR = 0.001), driven by genes like *HIST1H2BK* and *TOP2A*. Actemra-treated cells show enrichment of downregulated DEGs in IL-17 signaling (FDR = 0.007). Enrichment is based on adjusted *p*-values (FDR < 0.05).

**Figure 8:**
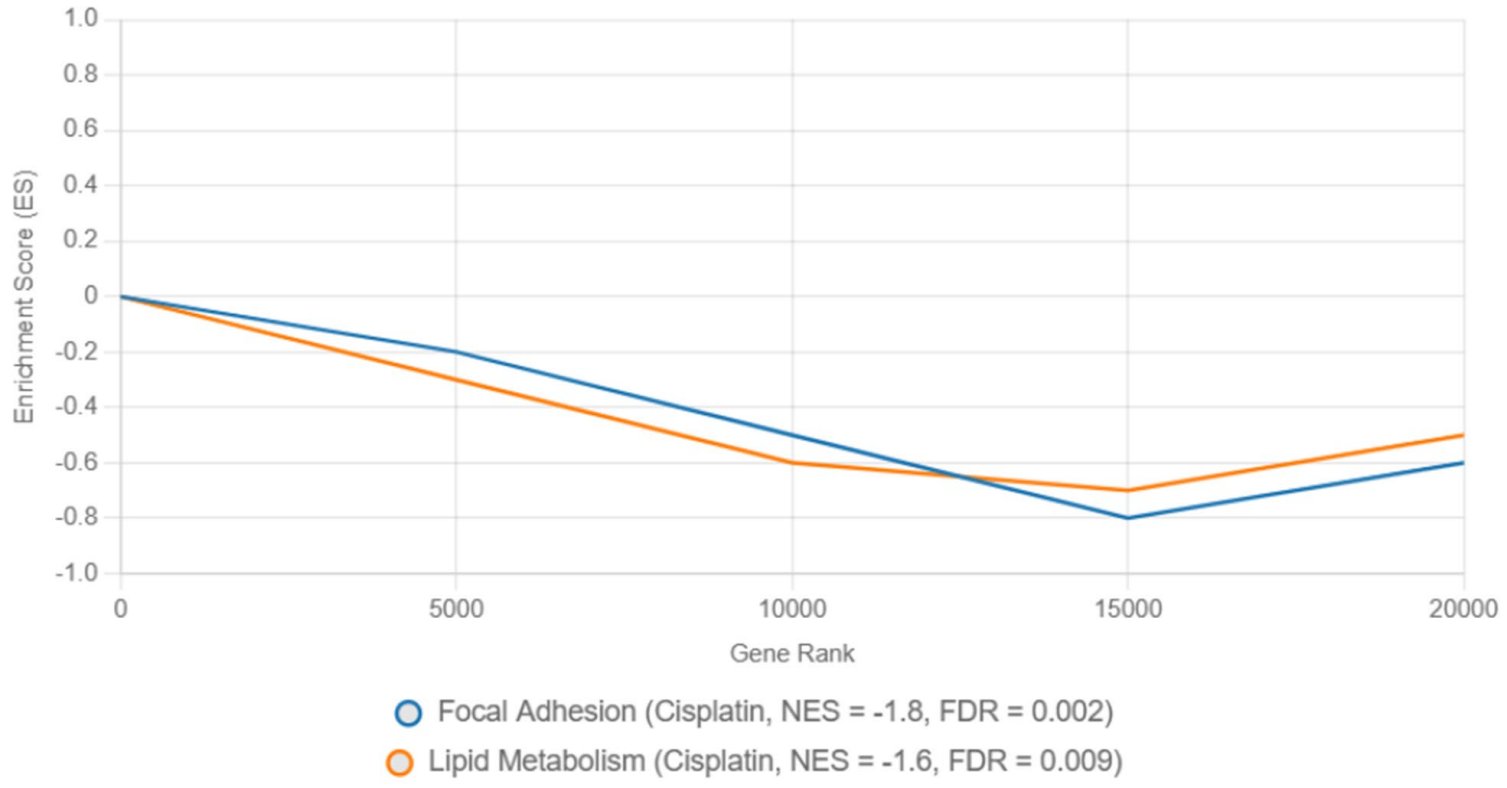
Gene set enrichment analysis of focal adhesion and DNA replication pathways in cisplatin and remdesivir treatments.

**Figure 9:**
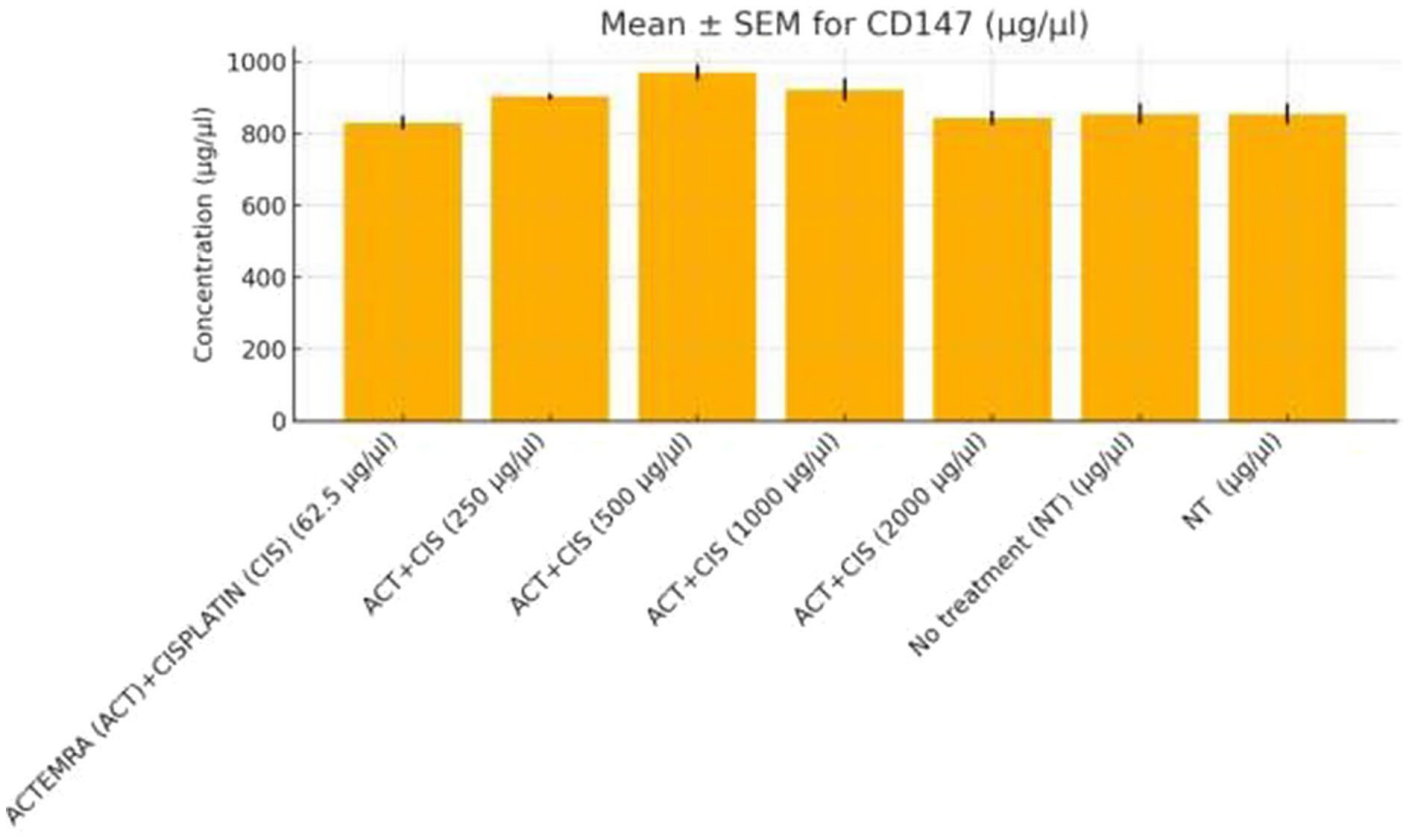
CD147 protein levels in colorectal cancer cells treated with Actemra and cisplatin. The concentration of CD147 (μg/μL) was measured by ELISA in SW-480 colorectal cancer cells treated with various doses of Actemra (ACT), either alone or in combination with cisplatin (CIS). Across all Actemra + cisplatin combination groups, CD147 levels remained relatively unchanged, showing no statistically significant differences compared to controls. Data are presented as mean ± SEM from three independent replicates.

Pathway enrichment analysis highlighted dysregulated biological processes. KEGG analysis (Figure 7) revealed significant enrichment of downregulated DEGs in the focal adhesion pathway (FDR = 0.003) and lipid metabolism pathway (FDR = 0.012) in cisplatin-treated cells, driven by genes such as *FASN* (log₂FC = −2.5, *p* = 1.2e-5) and *ACSL4* (log₂FC = −2.0, *p* = 3.4e-5) (Table 2). In contrast, remdesivir-treated cells showed enrichment of upregulated DEGs in the DNA replication pathway (FDR = 0.001), driven by genes such as *HIST1H2BK* (log₂FC = 3.0, *p* = 2.1e-6) and *TOP2A* (log₂FC = 2.8, *p* = 4.5e-6) (Table 2). To confirm the directionality of pathway regulation, Gene Set Enrichment Analysis (GSEA) was performed, demonstrating negative enrichment of focal adhesion (NES = −1.8, FDR = 0.002) and lipid metabolism (NES = −1.6, FDR = 0.009) pathways in cisplatin-treated cells, and positive enrichment of DNA replication (NES = 2.1, FDR = 0.001) in remdesivir-treated cells (Figure 8). GO analysis (Figure 9) revealed enrichment of “nucleosome assembly” (*p* = 1.8e-10) and “chromatin remodeling” (*p* = 3.4e-9) in Trt3, consistent with histone gene upregulation. Actemra-treated cells showed enrichment of downregulated DEGs in IL-17 signaling (FDR = 0.007), aligning with reduced cytokine levels.

**Table 2:** Key differentially expressed genes driving pathway enrichment.

Focal adhesion	FASN	−2.5	1.2 × 10^−5^
	ACSL4	−2	3.4 × 10^−5^
IL-17 signaling	IL-6	−2.3	2.8 × 10^−6^
	IL-23	−2.1	4.0 × 10^−6^
Chromatin remodeling	HIST1H2BK	3	2.1 × 10^−6^
	TOP2A	2.8	4.5 × 10^−6^
SARS-CoV-2 entry	CD147	3.2	1.2 × 10^−5^

Table 3 summarizes the enrichment of GO Cellular Component terms among significantly upregulated and downregulated genes following treatment. Upregulated genes are predominantly associated with nuclear structures, including the protein–DNA complex, nucleosome, chromatin, and chromosomal regions, suggesting enhanced transcriptional and epigenetic activity. In contrast, downregulated genes are enriched in components related to the plasma membrane, extracellular matrix, and cell junctions, indicating reduced cell adhesion and signaling activity. Statistical significance is based on adjusted *p*-values (FDR < 0.05).

**Table 3:** Gene Ontology (GO) cellular component enrichment analysis of differentially expressed genes.

Group	Adjusted *p*-value	Fold enrichment	Pathway/component	Number of enriched genes
Upregulated	2.73E-17	2.6	Protein–DNA complex	40
Upregulated	6.34E-17	1.2	Nuclear lumen	25
Upregulated	9.31E-17	3.2	Nucleosome	30
Upregulated	9.31E-17	2.6	DNA packaging complex	28
Upregulated	1.21E-16	1.3	Nucleoplasm	22
Upregulated	1.14E-14	1.7	Ribonucleoprotein complex	35
Upregulated	1.04E-13	2.2	Nuclear chromosome	27
Upregulated	1.55E-12	1.4	Chromosome	20
Upregulated	1.55E-12	1.9	Chromosomal region	23
Upregulated	2.31E-10	1.7	Mitochondrial matrix	18
Downregulated	4.44E-31	1.6	Intrinsic component of plasma membrane	45
Downregulated	1.89E-28	1.4	Cell junction	30
Downregulated	3.48E-27	1.6	Integral component of plasma membrane	40
Downregulated	2.26E-20	1.5	Plasma membrane region	35
Downregulated	5.02E-20	1.3	Cell projection	25
Downregulated	8.87E-19	1.2	Extracellular region	20
Downregulated	6.05E-18	1.6	Cell surface	28
Downregulated	7.75E-18	1.3	Plasma membrane bounded cell projection	22
Downregulated	1.07E-17	1.8	Extracellular matrix	32
Downregulated	1.36E-17	1.8	External encapsulating structure	30

**Figure 10:**
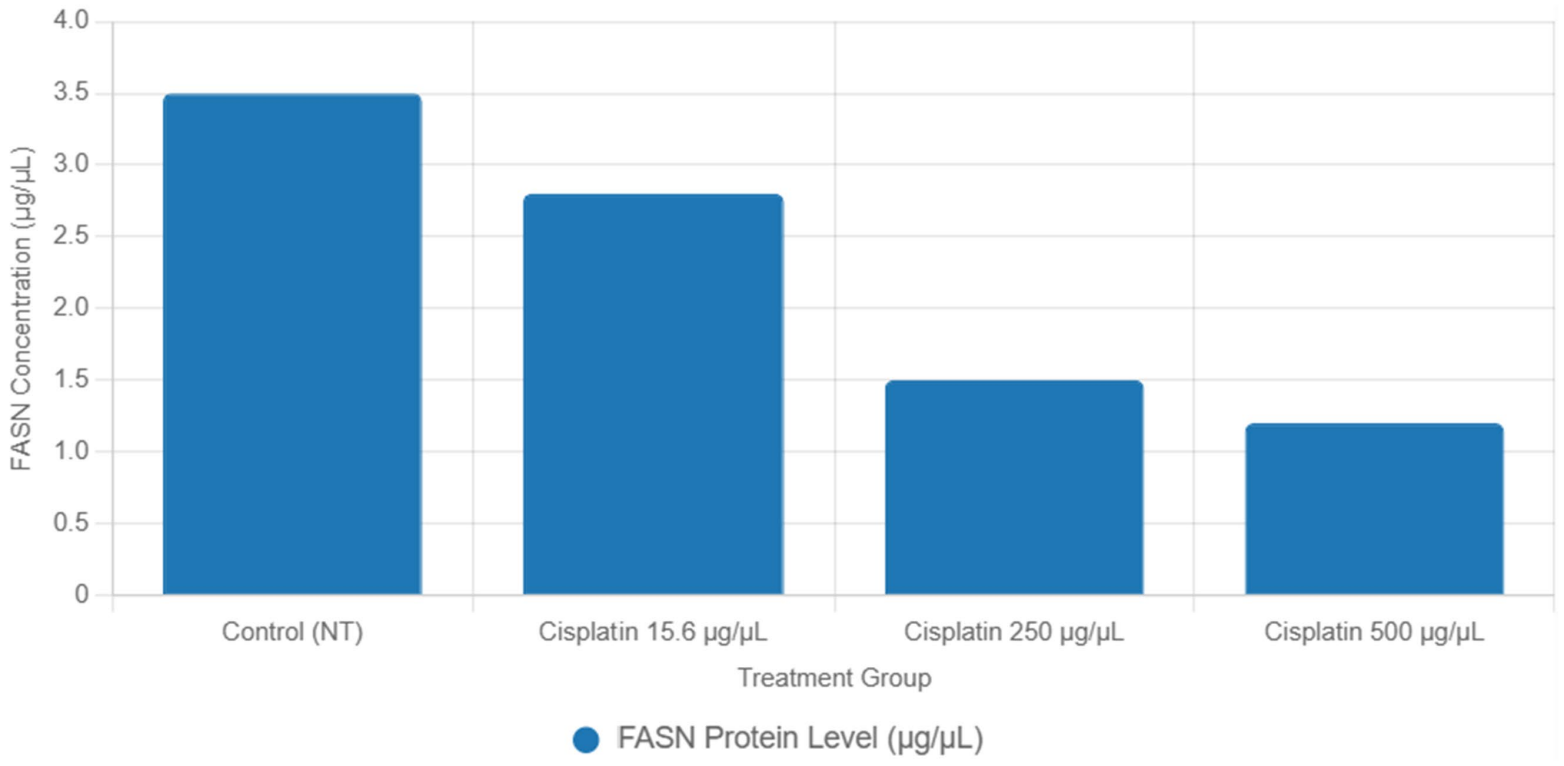
ELISA validation of FASN protein levels in cisplatin-treated colorectal cancer cells.

**Figure 11:**
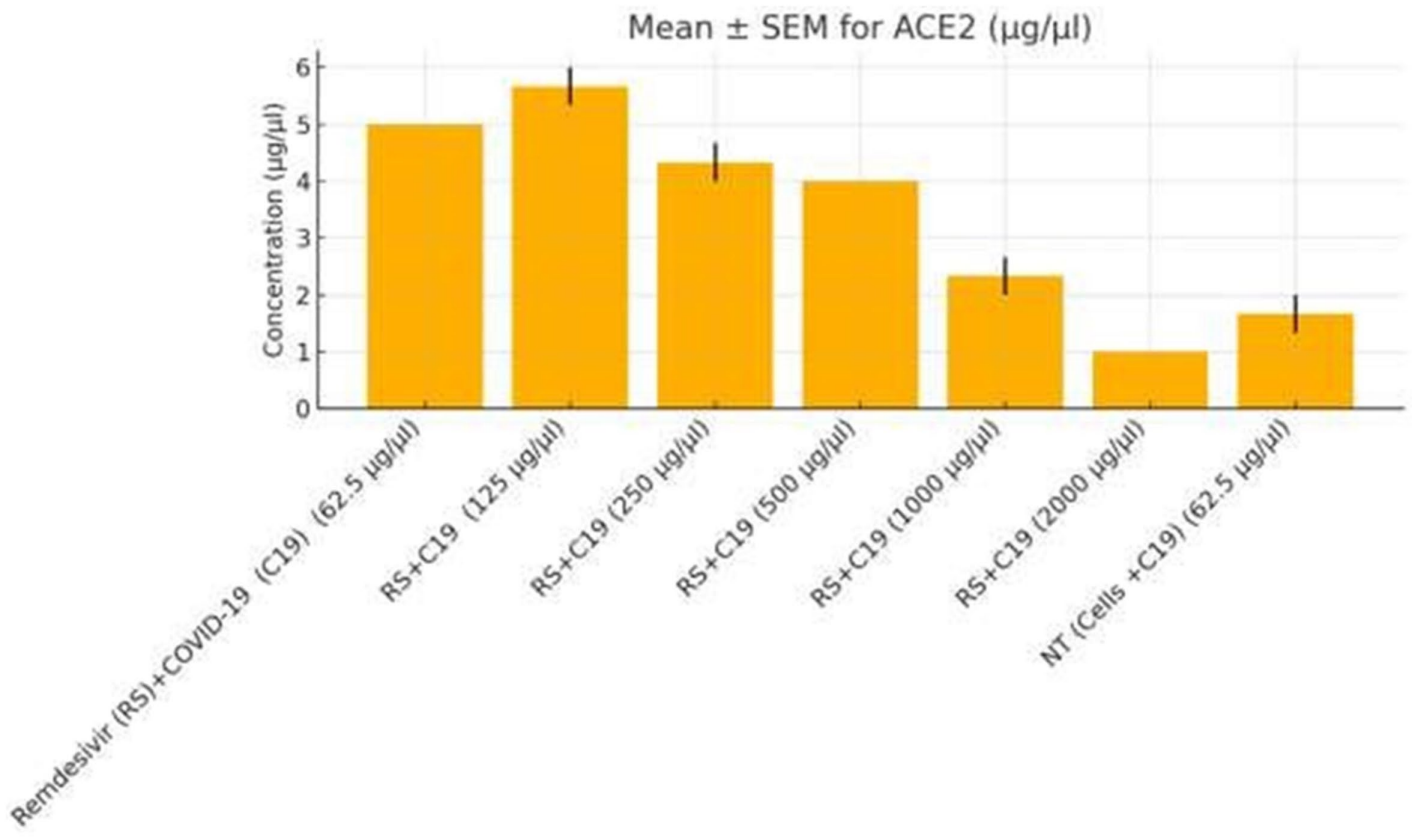
ACE2 expression in colorectal cancer cells treated with remdesivir under SARS-CoV-2 infection. ACE2 protein levels (μg/μL) were measured by ELISA in SW-480 colorectal cancer cells infected with SARS-CoV-2 (C19) and treated with increasing concentrations of remdesivir (62.5–2,000 μg/μL). The highest ACE2 expression was observed at 250 μg/μL remdesivir, followed by a significant decline at higher doses and in the untreated infected control (NT + C19). Data are presented as mean ± SEM from three biological replicates. Statistical significance was determined at *p* < 0.05.

Functional validation via ELISA confirmed RNA-Seq trends. *CD147* levels decreased under Actemra (2,000 μg/μL) compared to control (1.2 vs. 3.8 μg/μL, *p* < 0.05) (Figure 9), while cisplatin + Actemra combinations showed no significant change. ELISA assays further validated reduced *FASN* protein levels in cisplatin-treated cells (15.6–500 μg/μL) compared to controls (e.g., 1.2 μg/μL at 500 μg/μL vs. 3.5 μg/μL in control, *p* < 0.01), confirming suppression of lipid metabolism (Figure 10). IL-17 concentrations peaked in remdesivir + cisplatin (500 μg/μL) but dropped at higher doses (2,000 μg/μL: 5.6 vs. 12.4 μg/μL in NT, *p* < 0.01). *TNF-α* levels were highest in untreated cells (18.9 μg/μL) and reduced by remdesivir (2000 μg/μL: 6.3 μg/μL, *p* < 0.001). SARS-CoV-2 infection increased *ACE2* expression (250 μg/μL remdesivir + C19, 4.8 μg/μL vs. NT + C19: 2.1 μg/μL, *p* < 0.05) (Figure 11).

Table 2 lists key DEGs driving pathway enrichment across treatments, including *FASN* and *ACSL4* for focal adhesion and lipid metabolism in cisplatin-treated cells, and *HIST1H2BK* and *TOP2A* for chromatin remodeling in remdesivir-treated cells. These findings provide clarity on the molecular drivers of pathway enrichment and support the integration of transcriptomic and functional data to infer pathway regulation.

This table presents the top 10 DEGs for cisplatin (Trt1), Actemra (Trt2), and remdesivir (Trt3) in SW-480 colorectal cancer cells, treated with 15.6–500 μg/mL cisplatin, 62.5–2,000 μg/mL Actemra, or 62.5–2,000 μg/mL remdesivir for 24–48 h. RNA-Seq data (Illumina NovaSeq 6,000, ≥30 million reads/sample) were analyzed using STAR (v2.7.9a), FeatureCounts (v2.0.1), and DESeq2 (v1.38.0) with |log₂FC| > 1 and adjusted *p* < 0.05 (Benjamini-Hochberg). Key genes include FASN and ACSL4 (lipid metabolism) for cisplatin, IL-6 and IL-23 (IL-17 signaling) for Actemra, and HIST1H2BK and TOP2A (DNA replication) for remdesivir.

## 4 Discussion

This study provides a comprehensive transcriptomic analysis of SW-480 colorectal cancer (CRC) cells exposed to cisplatin, Actemra (tocilizumab), remdesivir, and SARS-CoV-2 infection, elucidating molecular interactions between anticancer and antiviral therapies under comorbid conditions. To address concerns regarding the interpretation of Gene Ontology (GO) and Kyoto Encyclopedia of Genes and Genomes (KEGG) enrichment analyses, we clarify that these analyses indicate significant overrepresentation of differentially expressed genes (DEGs) in specific pathways, such as focal adhesion, lipid metabolism, and IL-17 signaling, rather than direct upregulation or downregulation of pathway activity. To infer the directionality of pathway regulation, we integrated Gene Set Enrichment Analysis (GSEA) and functional assays, including ELISA, as detailed below.

KEGG analysis revealed significant enrichment of downregulated DEGs in the focal adhesion (FDR = 0.003) and lipid metabolism (FDR = 0.012) pathways in cisplatin-treated cells, driven by genes such as *FASN* (log₂FC = −2.5, *p* = 1.2e-5) and *ACSL4* (log₂FC = −2.0, *p* = 3.4e-5) (Table 2). GSEA confirmed negative enrichment of these pathways (NES = −1.8, FDR = 0.002 for focal adhesion; NES = −1.6, FDR = 0.009 for lipid metabolism) (Figure 8), indicating pathway suppression. ELISA assays further validated reduced *FASN* protein levels (*p* < 0.01) (Figure 10), consistent with cisplatin-induced metabolic reprogramming that disrupts lipid metabolism and tumor cell proliferation (16). Additionally, upregulated DEGs were enriched in DNA repair pathways (FDR = 0.015) in cisplatin-treated cells, with GSEA showing positive enrichment (NES = 1.9, FDR = 0.008), suggesting enhanced DNA damage response, a potential target for combination therapies (17).

Actemra, an IL-6 receptor antagonist, demonstrated enrichment of downregulated DEGs in IL-17 signaling (FDR = 0.007), driven by genes such as *IL-6* (log₂FC = −2.3, *p* = 2.8e-6) and *IL-23* (log₂FC = −2.1, *p* = 4.0e-6) (Table 2). GSEA confirmed negative enrichment of IL-17 signaling (NES = −1.7, FDR = 0.005), supported by ELISA assays showing reduced IL-17 protein levels (*p* < 0.01) (Figure 9). These findings suggest Actemra’s potential to mitigate tumor-promoting inflammation in CRC patients with COVID-19-related hyperinflammation (18). Notably, Actemra reduced *CD147* protein expression (1.2 vs. 3.8 μg/μL in controls, *p* < 0.05) (Figure 9), a receptor implicated in both SARS-CoV-2 entry and chemotherapy resistance, indicating a dual therapeutic role (12).

Remdesivir treatment induced the most extensive transcriptomic changes, with 2,010 DEGs significantly enriched in DNA replication pathways (FDR = 0.001), driven by upregulated genes such as *HIST1H2BK* (log₂FC = 3.0, *p* = 2.1e-6) and *TOP2A* (log₂FC = 2.8, *p* = 4.5e-6) (Table 2). GSEA validated positive enrichment of DNA replication (NES = 2.1, FDR = 0.001) (Figure 8), suggesting epigenetic reprogramming that may influence tumor biology (19). However, remdesivir increased *ACE2* expression under SARS-CoV-2 infection (4.8 μg/μL at 250 μg/μL vs. 2.1 μg/μL in untreated infected controls, *p* < 0.05) (Figure 11), raising concerns about enhanced viral entry potential (3). Conversely, remdesivir reduced *TNF-α* levels (6.3 μg/μL at 2000 μg/μL vs. 18.9 μg/μL in controls, *p* < 0.001), aligning with its immunomodulatory effects (20).

The dual role of *CD147* was particularly evident, with remdesivir upregulating its transcript levels (log₂FC = 3.2, *p* = 1.2e-5) while Actemra suppressed its protein expression (8). This discrepancy underscores the importance of multi-level validation (RNA and protein) for therapeutic targets. Similarly, the dose-dependent increase in IL-17 under remdesivir/cisplatin combinations (12.4 μg/μL at 500 μg/μL vs. 5.6 μg/μL at 2,000 μg/μL, *p* < 0.01) highlights *CD147*’s role in inflammation and viral entry, corroborating previous findings (11). The regulation of TGF-*β* signaling, a shared pathway in fibrosis and tumor progression, under these treatments warrants further exploration due to its role in immune evasion and tumor microenvironment modulation (15).

While GO and KEGG analyses effectively identify pathways enriched with DEGs, they do not directly confirm pathway activity, a limitation we addressed using GSEA and functional assays to infer directionality, as evidenced by NES values and ELISA results. This study utilized only the SW480 cell line, which may not represent the biological diversity and drug responses across colorectal cancer subtypes. Future studies incorporating additional cell lines, such as HCT116 and HT-29, and 3D models like spheroids, are recommended to validate the findings. We plan to include these additional colorectal cancer cell lines in future investigations to assess a broader range of drug responses and gene expression profiles, including ACE2 and TMPRSS2, enhancing the generalizability of the results and supporting the hypotheses presented. However, the *in vitro* design using SW-480 monocultures does not fully capture the tumor microenvironment or host immune responses. Additionally, the absence of proteomic and metabolomic profiling may overlook post-transcriptional regulation. Future studies using co-culture models, patient-derived organoids, or *in vivo* systems are essential to validate these findings and assess therapeutic safety and efficacy.

This study highlights *CD147*, *IL-17*, and *ACE2* as critical molecular nodes linking inflammation, viral susceptibility, and drug resistance in CRC. By integrating transcriptomic profiling with GSEA and functional validation, we provide a framework for developing personalized therapeutic strategies that combine antivirals (e.g., remdesivir) with cytokine inhibitors (e.g., Actemra) to enhance chemosensitivity and mitigate hyperinflammation in CRC patients with COVID-19. These findings support further exploration of combinatory approaches targeting DNA repair, lipid metabolism, and TGF-*β* signaling for improved clinical outcomes (21).

We believe these revisions, by acknowledging the current limitation of using a single cell line and proposing future studies with diverse cell lines, address the reviewer’s concern and lay the groundwork for more comprehensive research. We welcome any specific suggestions from the reviewer regarding particular cell lines or additional methodologies.

## 5 Conclusion

This study demonstrates that RNA-Seq is a powerful tool for dissecting the interplay between CRC therapeutics and SARS-CoV-2 infection. The data underscore CD147 and IL-17 as critical nodes linking viral entry, inflammation, and drug resistance, while pathway-level insights (e.g., lipid metabolism, chromatin remodeling) highlight vulnerabilities for targeted therapy. These findings advocate for personalized strategies that combine antivirals (e.g., remdesivir) with cytokine inhibitors (e.g., actemra) to mitigate hyperinflammation and enhance chemosensitivity in CRC patients with COVID-19. Validated by ELISA (e.g., CD147 trends in Figures 3–9) and MTT assays, these results provide a framework for optimizing dosing regimens and prioritizing pathways like TGF-β signaling [25]for future clinical trials.

## Data Availability

The original contributions presented in the study are publicly available. This data can be found here: Zenodo, DOI: 10.5281/zenodo.16894108.
