# Correction: RNA sequencing reveals transcriptional signatures of drug response and SARS-CoV-2 interaction in colorectal cancer cells

**DOI:** 10.3389/fmed.2026.1858713

**Published:** 2026-05-18

**Authors:** Alaa K. Hameed, Ifad K. Abd Al-Shibly, Rana A. Ghaleb

**Affiliations:** 1 Department of Medical Laboratories, College of Health & Medical Technology, Sawa University, Almuthana, Iraq; 2 Department of Microbiology, College of Medicine, Babylon University, Hilla, Iraq; 3 Department of Human Anatomy, College of Medicine, Babylon University, Hilla, Iraq

**Keywords:** RNA sequencing, colorectal cancer, cisplatin, Actemra, remdesivir, SARS-CoV-2

In the published article, the affiliation “Department of Medical Laboratories, College of Health & Medical Technology, Sawa University, Almuthana, Iraq” was erroneously given as “Department of Laboratories, Al-Hussein Teaching Hospital, Al-Muthanna Health Department Employee, Al-Muthanna, Iraq”.

The original version of this article has been updated.

