# Supplementary material for: RNA sequencing reveals transcriptional signatures of drug response and SARS-CoV-2 interaction in colorectal cancer cells
Source: Front Med (Lausanne). 2025 Sep 18;12:1654555. doi: 10.3389/fmed.2025.1654555 (PMC12488652; doi:10.3389/fmed.2025.1654555)
Supplement: Supplementary file 1 [file Supplementary_file_1.zip › SW480_Cnt1_2_fastqc/fastqc_report.html]

SW480\_Cnt1\_2.fastq FastQC Report 

FastQC Report

Sat 1 Feb 2025  
SW480\_Cnt1\_2.fastq

## Summary

- Basic Statistics
- Per base sequence quality
- Per tile sequence quality
- Per sequence quality scores
- Per base sequence content
- Per sequence GC content
- Per base N content
- Sequence Length Distribution
- Sequence Duplication Levels
- Overrepresented sequences
- Adapter Content

## Basic Statistics

| Measure | Value |
| --- | --- |
| Filename | SW480\_Cnt1\_2.fastq |
| File type | Conventional base calls |
| Encoding | Sanger / Illumina 1.9 |
| Total Sequences | 7011989 |
| Sequences flagged as poor quality | 0 |
| Sequence length | 101 |
| %GC | 57 |

## Per base sequence quality

## Per tile sequence quality

## Per sequence quality scores

## Per base sequence content

## Per sequence GC content

## Per base N content

## Sequence Length Distribution

## Sequence Duplication Levels

## Overrepresented sequences

| Sequence | Count | Percentage | Possible Source |
| --- | --- | --- | --- |
| GGGCGATCTGGCTGCGACATCTGTCACCCCATTGATCGCCAGGGTTGATT | 12705 | 0.18118967385716095 | No Hit |
| GGGAAGCTCATCAGTGGGGCCACGAGCTGAGTGCGTCCTGTCACTCCACT | 11694 | 0.16677151090796064 | No Hit |
| GGCGATCTGGCTGCGACATCTGTCACCCCATTGATCGCCAGGGTTGATTC | 9605 | 0.13697967866178912 | No Hit |
| CTGGGCTGTAGTGCGCTATGCCGATCGGGTGTCCGCACTAAGTTCGGCAT | 7599 | 0.10837153338375174 | No Hit |
| GTCGGGGGAGCGCGTCCCGGTCGCCGCGGTTCGCCGCCCGCCCCCGGTGG | 7061 | 0.10069896002403882 | No Hit |

## Adapter Content

Produced by FastQC (version 0.11.9)
