# Supplementary figures and images for: RNA sequencing reveals transcriptional signatures of drug response and SARS-CoV-2 interaction in colorectal cancer cells
Source: Front Med (Lausanne). 2025 Sep 18;12:1654555. doi: 10.3389/fmed.2025.1654555 (PMC12488652; doi:10.3389/fmed.2025.1654555)

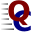

Supplement: Supplementary file 1 [file Supplementary_file_1.zip › SW480_Cnt1_2_fastqc/Icons/fastqc_icon.png]

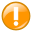

Supplement: Supplementary file 1 [file Supplementary_file_1.zip › SW480_Cnt1_2_fastqc/Icons/warning.png]

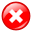

Supplement: Supplementary file 1 [file Supplementary_file_1.zip › SW480_Cnt1_2_fastqc/Icons/error.png]

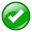

Supplement: Supplementary file 1 [file Supplementary_file_1.zip › SW480_Cnt1_2_fastqc/Icons/tick.png]

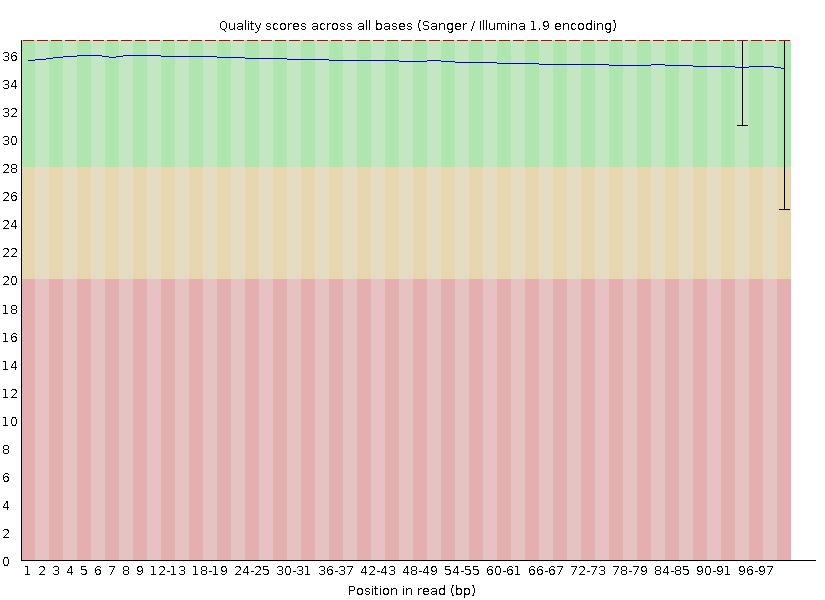

Supplement: Supplementary file 1 [file Supplementary_file_1.zip › SW480_Cnt1_2_fastqc/Images/per_base_quality.png]

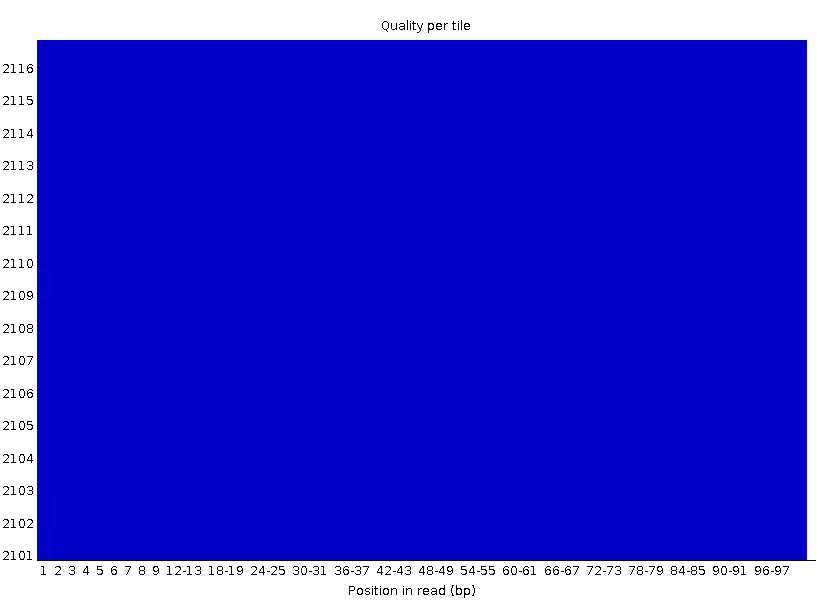

Supplement: Supplementary file 1 [file Supplementary_file_1.zip › SW480_Cnt1_2_fastqc/Images/per_tile_quality.png]

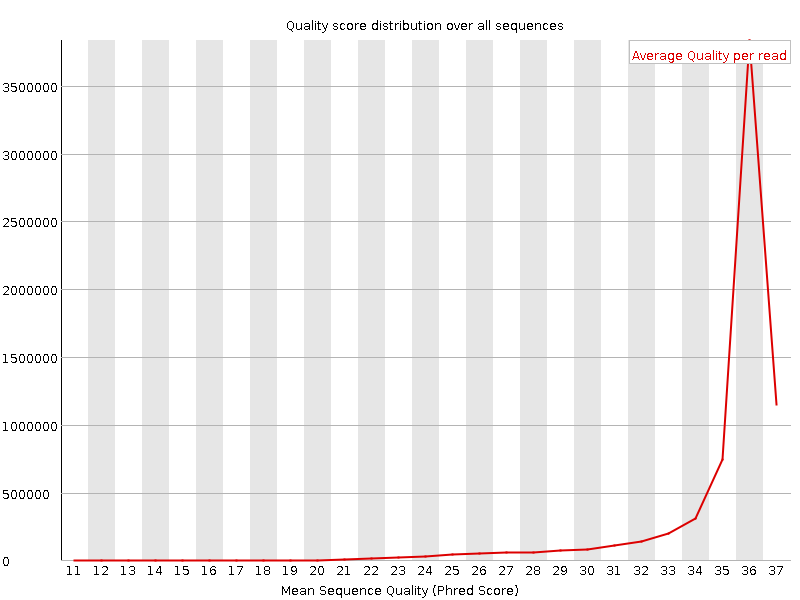

Supplement: Supplementary file 1 [file Supplementary_file_1.zip › SW480_Cnt1_2_fastqc/Images/per_sequence_quality.png]

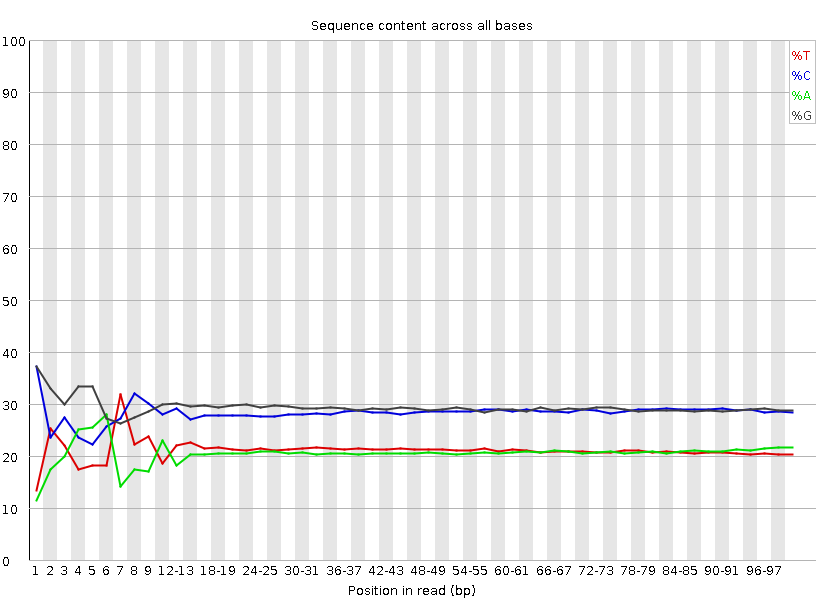

Supplement: Supplementary file 1 [file Supplementary_file_1.zip › SW480_Cnt1_2_fastqc/Images/per_base_sequence_content.png]

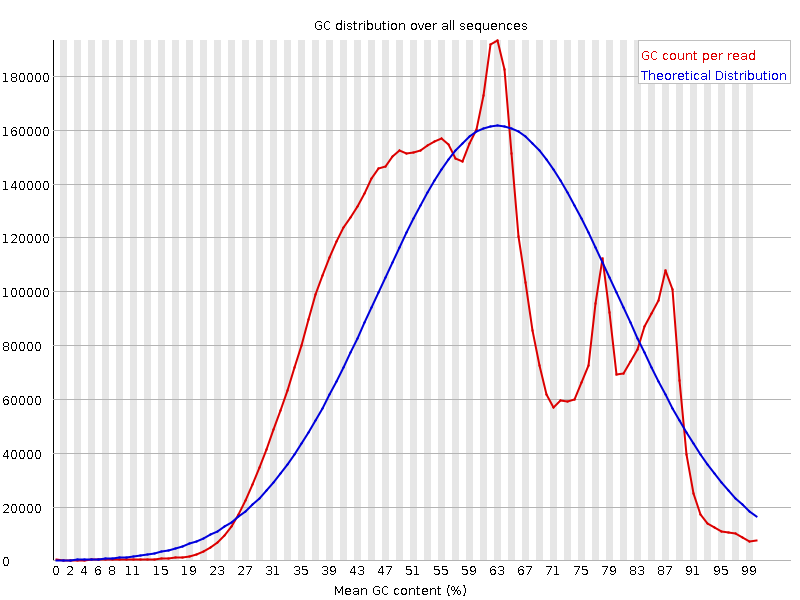

Supplement: Supplementary file 1 [file Supplementary_file_1.zip › SW480_Cnt1_2_fastqc/Images/per_sequence_gc_content.png]

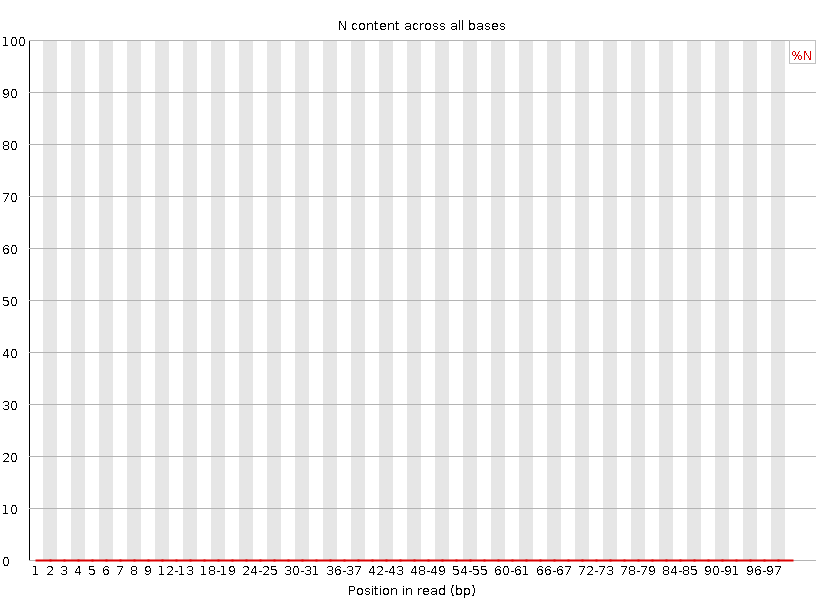

Supplement: Supplementary file 1 [file Supplementary_file_1.zip › SW480_Cnt1_2_fastqc/Images/per_base_n_content.png]

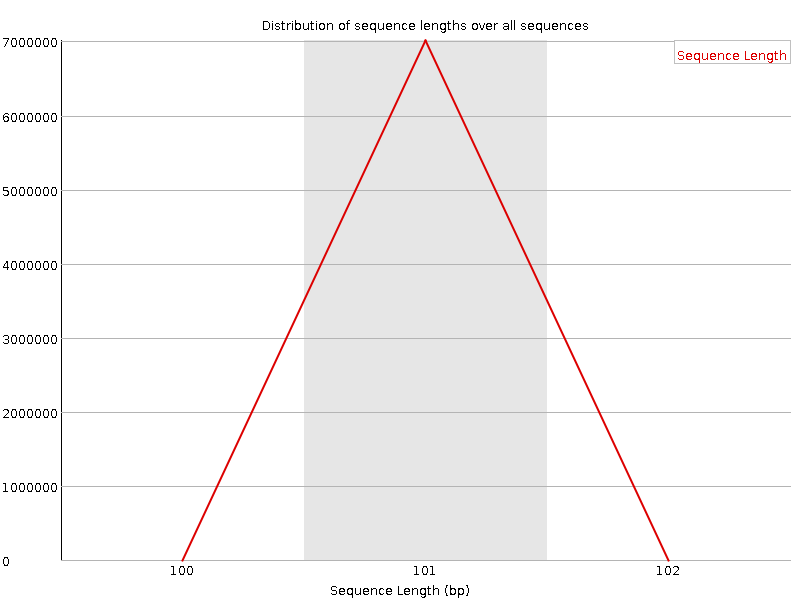

Supplement: Supplementary file 1 [file Supplementary_file_1.zip › SW480_Cnt1_2_fastqc/Images/sequence_length_distribution.png]

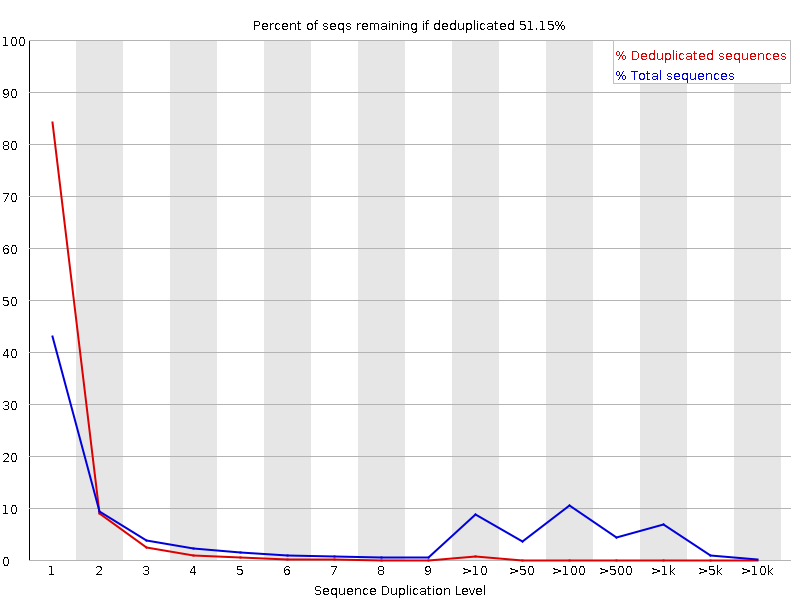

Supplement: Supplementary file 1 [file Supplementary_file_1.zip › SW480_Cnt1_2_fastqc/Images/duplication_levels.png]

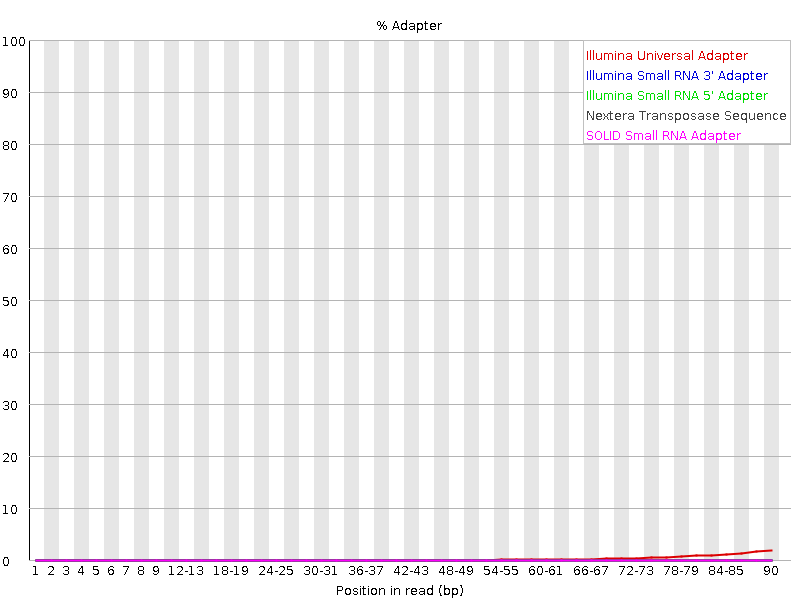

Supplement: Supplementary file 1 [file Supplementary_file_1.zip › SW480_Cnt1_2_fastqc/Images/adapter_content.png]

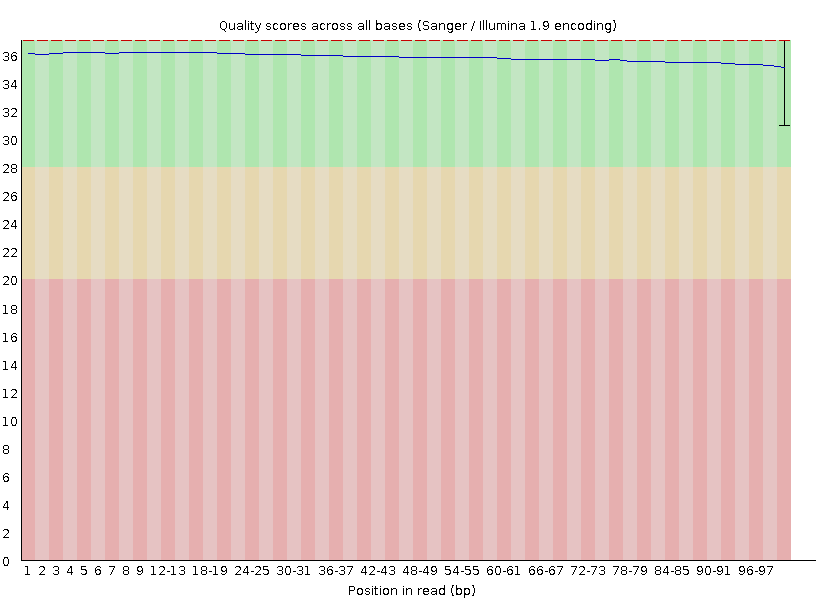

Supplement: Supplementary file 2 [file Supplementary_file_2.zip › SW480_Trt2_3_2_fastqc/Images/per_base_quality.png]

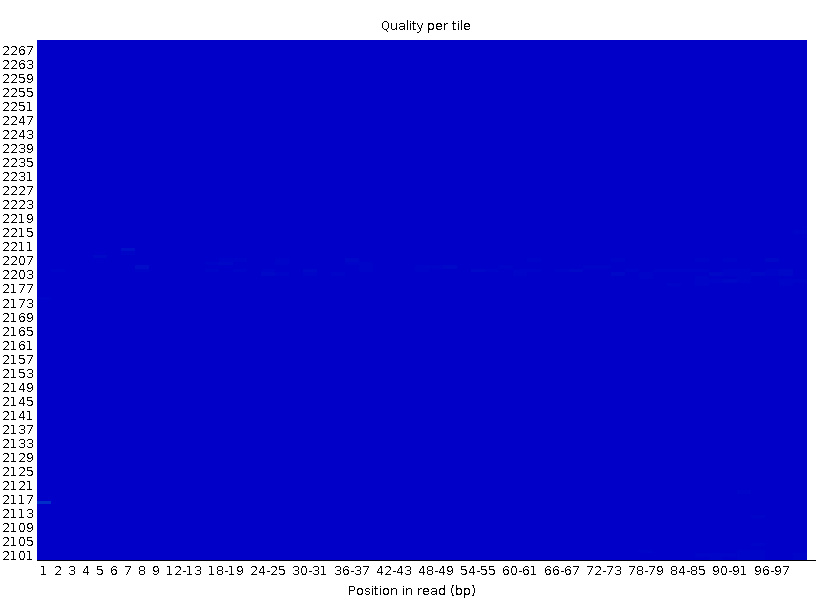

Supplement: Supplementary file 2 [file Supplementary_file_2.zip › SW480_Trt2_3_2_fastqc/Images/per_tile_quality.png]

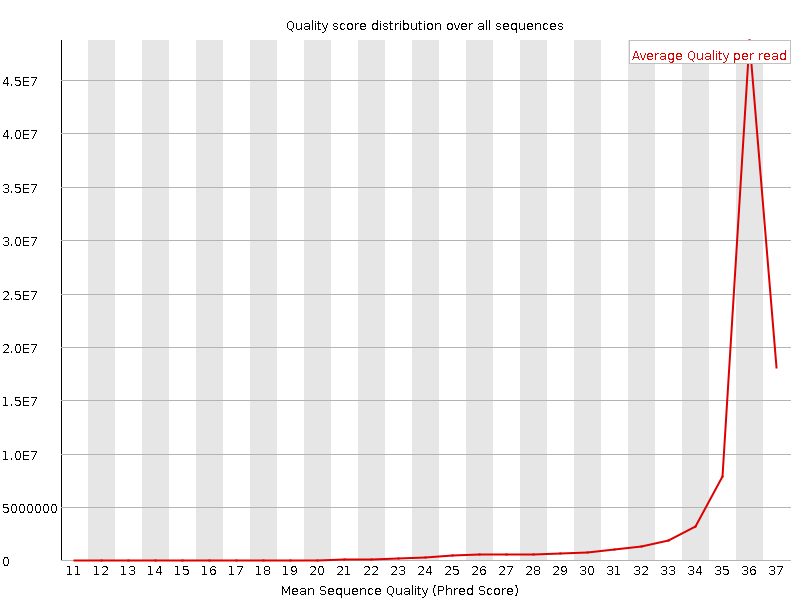

Supplement: Supplementary file 2 [file Supplementary_file_2.zip › SW480_Trt2_3_2_fastqc/Images/per_sequence_quality.png]

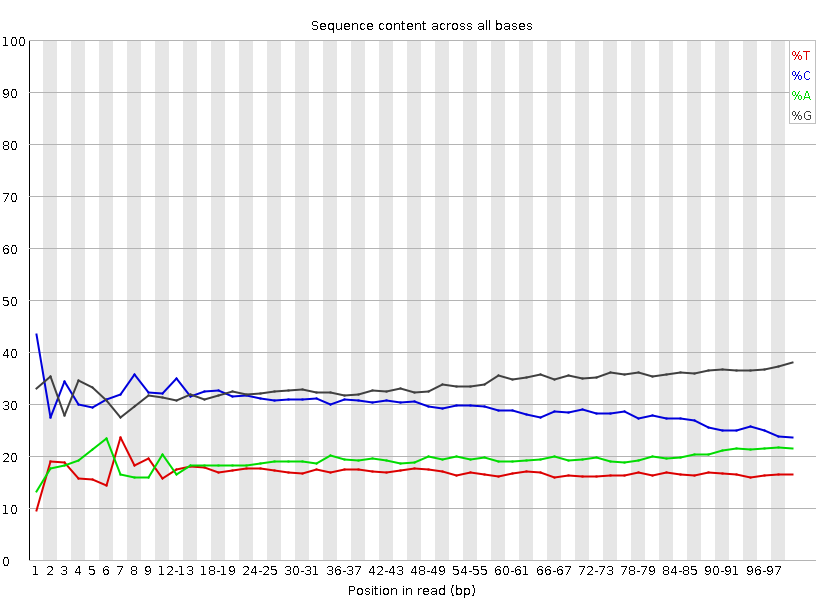

Supplement: Supplementary file 2 [file Supplementary_file_2.zip › SW480_Trt2_3_2_fastqc/Images/per_base_sequence_content.png]

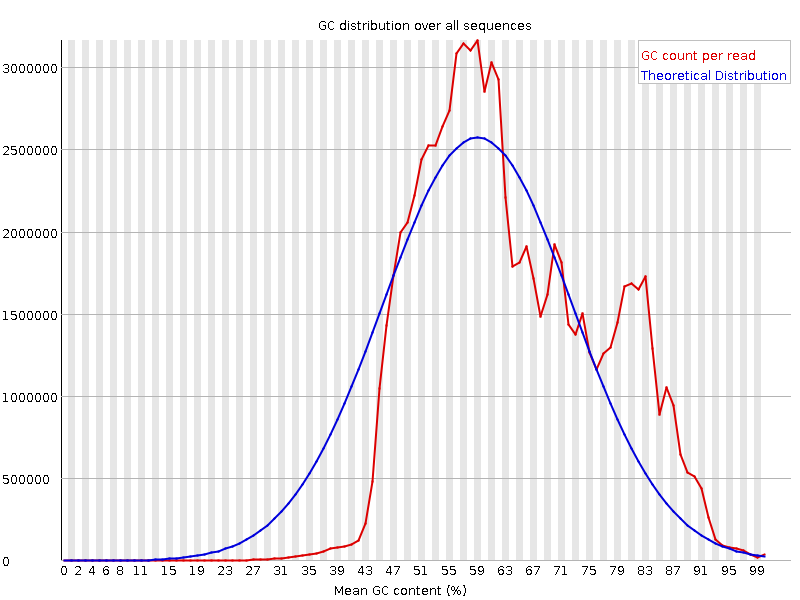

Supplement: Supplementary file 2 [file Supplementary_file_2.zip › SW480_Trt2_3_2_fastqc/Images/per_sequence_gc_content.png]

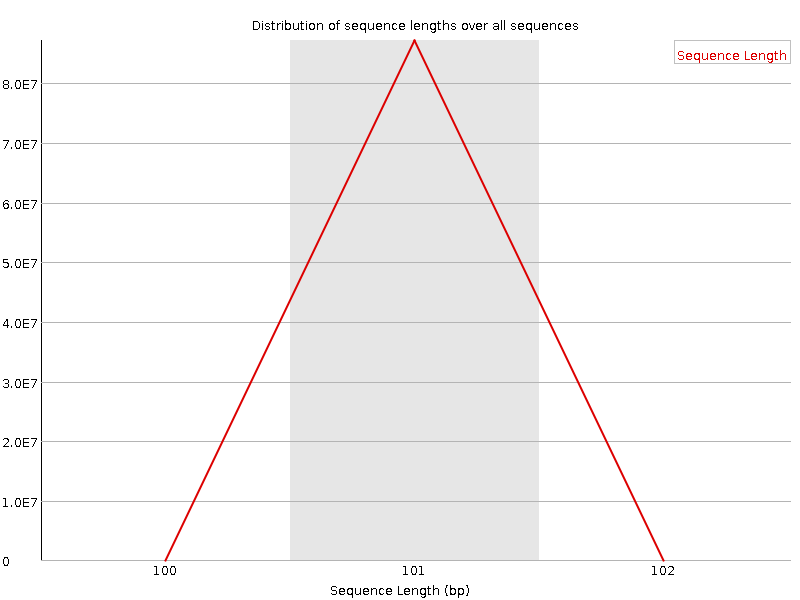

Supplement: Supplementary file 2 [file Supplementary_file_2.zip › SW480_Trt2_3_2_fastqc/Images/sequence_length_distribution.png]

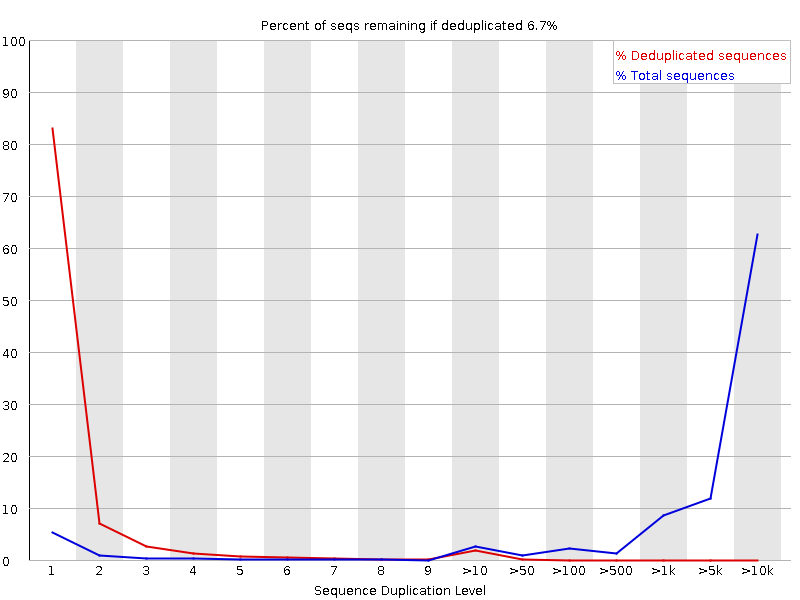

Supplement: Supplementary file 2 [file Supplementary_file_2.zip › SW480_Trt2_3_2_fastqc/Images/duplication_levels.png]

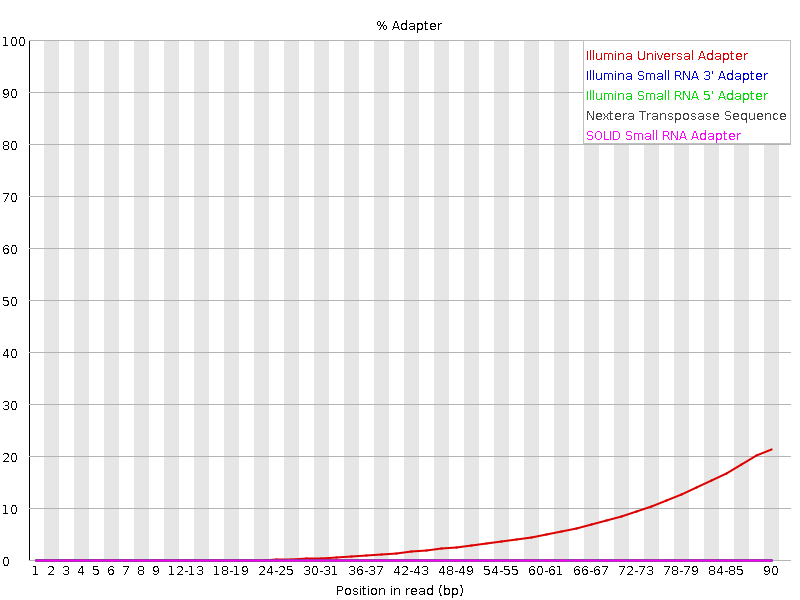

Supplement: Supplementary file 2 [file Supplementary_file_2.zip › SW480_Trt2_3_2_fastqc/Images/adapter_content.png]
