# Supplementary material for: RNA sequencing reveals transcriptional signatures of drug response and SARS-CoV-2 interaction in colorectal cancer cells
Source: Front Med (Lausanne). 2025 Sep 18;12:1654555. doi: 10.3389/fmed.2025.1654555 (PMC12488652; doi:10.3389/fmed.2025.1654555)
Supplement: Supplementary file 2 [file Supplementary_file_2.zip › SW480_Trt2_3_2_fastqc/fastqc_report.html]

SW480\_Trt2\_3\_2.fastq FastQC Report 

FastQC Report

Sat 1 Feb 2025  
SW480\_Trt2\_3\_2.fastq

## Summary

- Basic Statistics
- Per base sequence quality
- Per tile sequence quality
- Per sequence quality scores
- Per base sequence content
- Per sequence GC content
- Per base N content
- Sequence Length Distribution
- Sequence Duplication Levels
- Overrepresented sequences
- Adapter Content

## Basic Statistics

| Measure | Value |
| --- | --- |
| Filename | SW480\_Trt2\_3\_2.fastq |
| File type | Conventional base calls |
| Encoding | Sanger / Illumina 1.9 |
| Total Sequences | 87178925 |
| Sequences flagged as poor quality | 0 |
| Sequence length | 101 |
| %GC | 63 |

## Per base sequence quality

## Per tile sequence quality

## Per sequence quality scores

## Per base sequence content

## Per sequence GC content

## Per base N content

## Sequence Length Distribution

## Sequence Duplication Levels

## Overrepresented sequences

| Sequence | Count | Percentage | Possible Source |
| --- | --- | --- | --- |
| CCTGCCAGTAGCATATGCTTGTCTCAAAGATTAAGCCATGCATGTCTAAG | 448667 | 0.5146507599170327 | No Hit |
| CCTCACCCGGCCCGGACACGGACAGGATTGACAGATTGATAGCTCTTTCT | 390287 | 0.4476850339689323 | No Hit |
| GGAGTCTAACACGTGCGCGAGTCGGGGGCTCGCACGAAAGCCGCCGTGGC | 308866 | 0.35428975523614226 | No Hit |
| TCCTGCCAGTAGCATATGCTTGTCTCAAAGATTAAGCCATGCATGTCTAA | 233500 | 0.26783996246799324 | No Hit |
| AAGGAGTCTAACACGTGCGCGAGTCGGGGGCTCGCACGAAAGCCGCCGTG | 221488 | 0.25406140302831215 | No Hit |
| GACAGGTTAGTTTTACCCTACTGATGATGTGTTGTTGCCATGGTAATCCT | 188410 | 0.2161187465892703 | No Hit |
| CTCACCCGGCCCGGACACGGACAGGATTGACAGATTGATAGCTCTTTCTC | 167109 | 0.19168508902811088 | No Hit |
| GACTAATCGAACCATCTAGTAGCTGGTTCCCTCCGAAGTTTCCCTCAGGA | 165064 | 0.18933933860735264 | No Hit |
| CAGATCAGACGTGGCGACCCGCTGAATTTAAGCATATTAGTCAGCGGAGG | 161061 | 0.18474763252701268 | No Hit |
| GTCTGGTGCCAGCAGCCGCGGTAATTCCAGCTCCAATAGCGTATATTAAA | 158848 | 0.18220917498122394 | No Hit |
| GTTAGTTTTACCCTACTGATGATGTGTTGTTGCCATGGTAATCCTGCTCA | 154971 | 0.1777619992446569 | No Hit |
| CTCGGGCCGATCGCACGCCCCCCGTGGCGGCGACGACCCATTCGAACGTC | 140918 | 0.16164227764909925 | No Hit |
| GTAACAACTCACCTGCCGAATCAACTAGCCCTGAAAATGGATGGCGCTGG | 125780 | 0.14427798920438628 | No Hit |
| GTCGGAATCCGCTAAGGAGTGTGTAACAACTCACCTGCCGAATCAACTAG | 124254 | 0.14252756615202583 | No Hit |
| CTCTGGTGGAGGTCCGTAGCGGTCCTGACGTGCAAATCGGTCGTCCGACC | 117130 | 0.1343558663977561 | No Hit |
| CAACTTTCGATGGTAGTCGCCGTGCCTACCATGGTGACCACGGGTGACGG | 113506 | 0.13019889841495522 | No Hit |
| CGGGCGCTGACCCCCTTCGCGGGGGGGATGCGTGCATTTATCAGATCAAA | 113372 | 0.13004519154141897 | No Hit |
| GTCGGAGGTTCGAAGACGATCAGATACCGTCGTAGTTCCGACCATAAACG | 106796 | 0.12250208407593922 | No Hit |
| GTTTTACCCTACTGATGATGTGTTGTTGCCATGGTAATCCTGCTCAGTAC | 103746 | 0.11900353210365924 | No Hit |
| CTTTTGGTAAGCAGAACTGGCGCTGCGGGATGAACCGAACGCCGGGTTAA | 101046 | 0.1159064533085261 | No Hit |
| GGAGTGTGTAACAACTCACCTGCCGAATCAACTAGCCCTGAAAATGGATG | 100882 | 0.11571833444837729 | No Hit |
| GGACGGTGGCCATGGAAGTCGGAATCCGCTAAGGAGTGTGTAACAACTCA | 99399 | 0.11401723524349491 | No Hit |
| GTCTAACACGTGCGCGAGTCGGGGGCTCGCACGAAAGCCGCCGTGGCGCA | 98255 | 0.1127049914873348 | No Hit |
| CGCCCCGCTCCCCGCCCCCGGAGCCCCGCGGACGCTACGCCGCGACGAGT | 98121 | 0.11255128461379858 | No Hit |
| CTACTATCCAGCGAAACCACAGCCAAGGGAACGGGCTTGGCGGAATCAGC | 96854 | 0.11109795171252686 | No Hit |
| CCGCTAAGGAGTGTGTAACAACTCACCTGCCGAATCAACTAGCCCTGAAA | 94361 | 0.1082383156250206 | No Hit |
| GGCCGTTCTTAGTTGGTGGAGCGATTTGTCTGGTTAATTCCGATAACGAA | 93967 | 0.10778637153417527 | No Hit |
| GATCCCGAGGCCTCTCCAGTCCGCCGAGGGCGCACCACCGGCCCGTCTCG | 92166 | 0.10572050527119943 | No Hit |
| CAGAAACCTCCCGTGGAGCAGAAGGGCAAAAGCTCGCTTGATCTTGATTT | 90266 | 0.10354107945240207 | No Hit |
| CAAGGAGTCTAACACGTGCGCGAGTCGGGGGCTCGCACGAAAGCCGCCGT | 89334 | 0.10247201373497093 | No Hit |
| CTGGCTTGTGGCGGCCAAGCGTTCATAGCGACGTCGCTTTTTGATCCTTC | 89272 | 0.10240089562930489 | No Hit |
| CGAACGTCTGCCCTATCAACTTTCGATGGTAGTCGCCGTGCCTACCATGG | 89258 | 0.10238483670221904 | No Hit |
| CCCGACCCCTCCACCCGCCCTCCCTTCCCCCGCCGCCCCTCCTCCTCCTC | 89181 | 0.10229651260324671 | No Hit |
| CATGGGTCAGTCGGTCCTGAGAGATGGGCGAGCGCCGTTCCGAAGGGACG | 88915 | 0.10199139298861508 | No Hit |
| GGAAACCTCACCCGGCCCGGACACGGACAGGATTGACAGATTGATAGCTC | 88114 | 0.10107259294605893 | No Hit |
| CGCCTCCTCCCCTCCTCCCGCCCACGCCCCGCTCCCCGCCCCCGGAGCCC | 87600 | 0.1004830009087632 | No Hit |
| CCCCATTCGTGATGGGGATCGGGGATTGCAATTATTCCCCATGAACGAGG | 87294 | 0.1001319986453148 | No Hit |
| GGGAAGTCGGCAAGCCGGATCCGTAACTTCGGGATAAGGATTGGCTCTAA | 87230 | 0.10005858640720793 | No Hit |

## Adapter Content

Produced by FastQC (version 0.11.9)
